# Structural Engineering of Hierarchical Aerogels Comprised of Multi-dimensional Gradient Carbon Nanoarchitectures for Highly Efficient Microwave Absorption

**DOI:** 10.1007/s40820-021-00667-7

**Published:** 2021-06-15

**Authors:** Yongpeng Zhao, Xueqing Zuo, Yuan Guo, Hui Huang, Hao Zhang, Ting Wang, Ningxuan Wen, Huan Chen, Tianze Cong, Javid Muhammad, Xuan Yang, Xinnan Wang, Zeng Fan, Lujun Pan

**Affiliations:** 1 School of Physics, Dalian University of Technology, Dalian, 116024 Liaoning People’s Republic of China; 2 School of Microelectronics, Dalian University of Technology, Dalian, 116024 Liaoning People’s Republic of China; 3 School of Materials Science and Engineering, Dalian University of Technology, Dalian, 116024 Liaoning People’s Republic of China; 4 School of Chemical Engineering, Dalian University of Technology, Dalian, 116024 Liaoning People’s Republic of China

**Keywords:** Hierarchical aerogels, Multi-dimensional gradient, Carbon nanocoils, Microwave absorption

## Abstract

Recently, multilevel structural carbon aerogels are deemed as attractive candidates for microwave absorbing materials. Nevertheless, excessive stack and agglomeration for low-dimension carbon nanomaterials inducing impedance mismatch are significant challenges. Herein, the delicate “3D helix–2D sheet–1D fiber–0D dot” hierarchical aerogels have been successfully synthesized, for the first time, by sequential processes of hydrothermal self-assembly and in-situ chemical vapor deposition method. Particularly, the graphene sheets are uniformly intercalated by 3D helical carbon nanocoils, which give a feasible solution to the mentioned problem and endows the as-obtained aerogel with abundant porous structures and better dielectric properties. Moreover, by adjusting the content of 0D core–shell structured particles and the parameters for growth of the 1D carbon nanofibers, tunable electromagnetic properties and excellent impedance matching are achieved, which plays a vital role in the microwave absorption performance. As expected, the optimized aerogels harvest excellent performance, including broad effective bandwidth and strong reflection loss at low filling ratio and thin thickness. This work gives valuable guidance and inspiration for the design of hierarchical materials comprised of dimensional gradient structures, which holds great application potential for electromagnetic wave attenuation.

**Supplementary Information:**

The online version contains supplementary material available at 10.1007/s40820-021-00667-7.

## Highlights

- The delicate “3D helix–2D sheet–1D fiber–0D dot” hierarchical aerogels were successfully synthesized.
- The graphene sheets are uniformly intercalated by helical carbon nanocoils, which endow the as-obtained aerogel with abundant porous structures and better dielectric properties.
- By adjusting the growth parameters of 0D core-shell structured particles and 1D carbon nanofibers, the tunable electromagnetic properties and excellent impedance matching are achieved.

**Supplementary Information:**

The online version contains supplementary material available at 10.1007/s40820-021-00667-7.

## Introduction

Nowadays, microwave absorbing materials (MWAMs) have received great interest for their wide applications in wireless communications, information processing and Radar stealth technologies [1–4]. To date, a large variety of traditional materials have been developed to be used as MWAMs, such as transition metals (Fe [5], Co [6], Ni [7], Mn [8], and their alloys, ferrites, and ceramics [9, 10]). However, due to the large bulk densities, poor mechanical and chemical properties, the traditional materials hardly meet practical application requirements in future high-tech era. Thus, there has an urgent pursuit of advanced MWAMs with strong attenuation capability, wide effective bandwidth, low density, and thin thickness.

In general, microwave absorption performances of MWAMs are significantly linked with the parameters of complex permittivity and permeability of these materials. Therefore, an ingenious design for the special structures with tailorable electromagnetic parameters is a feasible strategy to upgrade their microwave absorption performance. Up to now, the porous [11–13], core-shell [14–18], yolk-shell [19, 20], and hierarchical-structured [21–28] materials have been certified as efficient MWAMs due to their special structures endowed with multiple microwave attenuation mechanisms. Among them, hierarchically structured materials are undoubtedly a kind of up-and-coming candidate for MWAMs due to their features of low density, multi-dimensional components, highly tunable dielectric loss, high specific surface area, and favorable impedance matching. For example, Che and his colleagues fabricated a 3D hierarchically structured MoS_2_/FeS_2_ composite, which showed an extended effective absorption bandwidth (EAB) of 6.48 GHz with a filler loading ratio of 50 wt% [29]. Liu et al. synthesized a composite of carbon fiber@MXene@MoS_2_, the minimum reflection loss (RL_min_) value reached − 61.5 dB at a thickness of 3.5 mm [30]. Wu and his co-authors prepared an efficient EMAM of hierarchical cobalt nanocrystals/N-doped carbon nanotubes (CNTs)/carbon sponge, the RL_min_ value reached − 52 dB, and the EAB was 4.1 GHz at the thickness of 2.2 mm [31]. Inspired by the excellent microwave absorption performances of these unique structures mentioned above, the novel architecture with hierarchical structure and tunable electromagnetic parameter is a promising way to upgrade the performance of EMAMs.

For decades, carbon-based microwave absorbing materials, such as 0D fullerene, 1D CNTs and nanofibers (CNFs), 2D graphene, and 3D carbon nanocoils (CNCs), have attracted tremendous attention due to their outstanding dielectric loss capacity, good corrosion resistance, high physical/chemical stability, and lightweight [32–34]. Meanwhile, it is worth noting that the differences in hybridization form of carbon atoms and morphology would affect the configurations and microwave absorbing properties of nanostructures. Typically, 0D structured materials (fullerene or carbon-based particles) have the advantage of integrating the magnetic and dielectric materials [35]. 1D structured materials (CNTs or CNFs) have the advantages of high anisotropy and aspect ratio, which facilitate the conducting loss during carrier transporting [36]. Besides, 2D graphene consumes energy through polarization loss and multiple reflection loss caused by large specific surface area, abundant oxygen-containing functional groups and multilayer structure [37]. Notably, the latest researches have reported that hierarchical structures comprising multi-dimensional carbon materials, such as the hybrid of particles, CNTs, and graphene, exhibit enhanced microwave absorption performances. However, the excessive stack of graphene and the agglomeration of CNTs/CNFs would reduce the porosity and interfacial compatibility of composite, which cause the interfacial impedance mismatching [2]. On the other hand, higher conductivities of graphitized carbon nanomaterials (CNT, CNF, and graphene) lead to skin effect that inhibits the further incidence of microwave into the EMAM. Therefore, it is very important to develop a promising method to tailor the conductivities of carbon-based hierarchical structures. To settle the tough challenges mentioned above, a novel structure and modified preparation strategy should be used. Interestingly, the CNCs give the new inspiration for designing novel composite materials due to its 3D morphology, chiral, and amorphous structure [38–42]. To date, the CNC-based composites have exhibited promising microwave attenuation capability. For example, Qin et al. prepared Fe_3_O_4_/Al_2_O_3_/CNCs by an atom layer deposition method, and the value of RL_min_ reached − 50.7 dB at the thickness of 2.4 mm [33]. Cui and co-authors prepared a CNCs/tissue composite, the RL_min_ value reached − 46.3 dB, and the EAB was 7.4 GHz at the thickness of 2 mm [43]. Recently, our group reported a core-shell structured Fe_3_O_4_@C/CNC absorber [44], and the *RL*_min_ reached − 47.5 dB at the thickness of 1.7 mm, while the value of EAB reached 5.03 GHz at the filler loading ratio of 40 wt%. On the other hand, the unique 3D helical skeleton would alleviate the stacking of graphene layers and boost the porosity of the composite. More importantly, the chiral CNCs would induce cross-polarization loss, which makes the material possess outstanding dielectric loss performance [45]. Therefore, considering the issue of excessive stack and agglomeration for low-dimension carbon materials, as well as the challenge of impedance mismatch caused by excessive conductivity, it is a highly promising way to introduce the 3D amorphous CNCs into the hierarchical structure for further improvements of energy attenuation and impedance matching.

Herein, we report a scalable strategy to fabricate hierarchical aerogels composed of CNCs/graphene/CNFs/ core-shell particles with the “3D helix–2D sheet–1D fiber–0D dot” structure by using self-assembly and in-situ chemical vapor deposition (CVD) methods. The advantages of this work are summarized as follow. First, the 2D graphene layer is evenly inserted by the 3D spiral CNCs, which gives the aerogel the porous structure and better dielectric properties. Then, two kinds of metal oxide nanoparticles (M-NPs) play different roles in the following CVD process. The NiO nanoparticle is used as a catalyst to synthesize 1D structured CNFs. As a result, the confined CNFs in aerogel tailor the conductive loss, which would significantly upgrade the dielectric loss of material. Besides, the 0D Fe_3_O_4_@C core-shell composites derived from Fe_2_O_3_ would not only endow the hybrid materials with the magnetic loss but also improve the impedance matching. Consequently, the as-prepared hierarchical structures deliver a strong attenuation capability and wide effective bandwidth at low filling ratio and thin thickness. This work would shed light on the delicate design of hierarchically structured aerogel for high-performance microwave absorption and other potential applications.

## Experimental Section

**Fig. 1:**
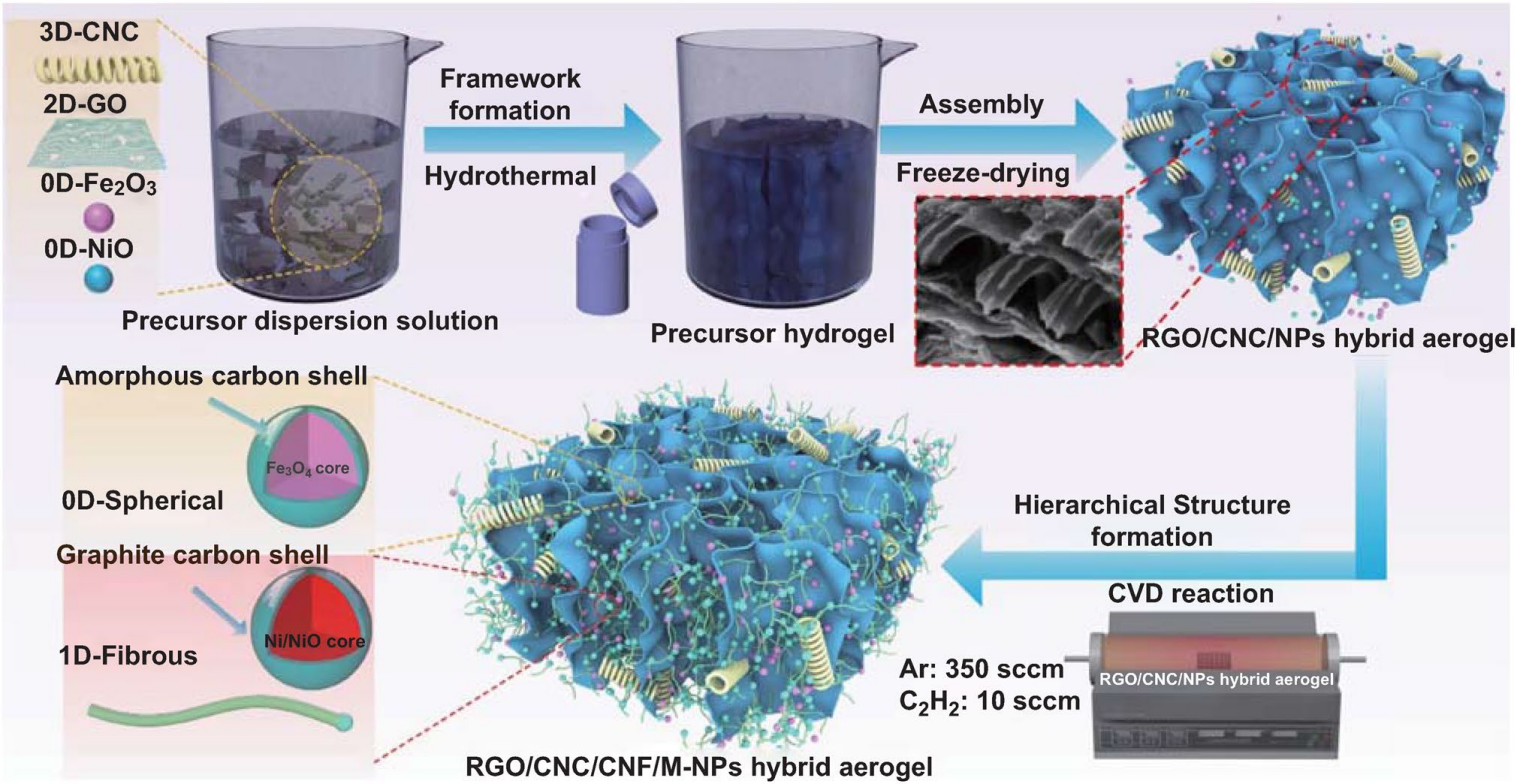
Overall synthesis procedure of the RGO/CNC/CNF/M-NPs hierarchical aerogel

The synthesis procedure of the hierarchical aerogel is schematically shown in Fig. 1. First, the reduced graphene oxide (RGO)/CNC/M-NPs aerogel was synthesized by a sequential process of hydrothermal and freeze-dry methods. Whereafter, the hierarchical RGO/CNC/CNF/M-NPs aerogels were obtained through the CVD process. By adjusting the CVD reaction time and the content of M-NPs, the samples with different electromagnetic properties were synthesized. A series of CNC-contained samples based on different synthesis conditions are labeled as GCA-M_*X*_–*Y* (*X* = 0.2, 0.3 mmol, and *Y* = 0, 5, 10, and 20 min, respectively). The subscript *X* in labels represents the total molar mass of M-NPs in the nanocomposite, while the *Y* represents reaction time of CVD process. The additional control samples were also fabricated without the addition of CNCs. Under this condition, the samples are designated as GA–M_*X*_–*Y*. For example, the sample GCA–M_0.2_–10 indicates the content of M-NPs in aerogel was 0.2 mmol, while the reaction time of the subsequent CVD process was 10 min. The details of each synthesis procedure were as follows.

### Materials

Graphene oxide powder was purchased from Nanjing XFNANO Materials. Besides, the CVD technology was used to synthesize high-purity CNCs by using porous Fe_2_O_3_/SnO_2_ nanoparticles as the catalyst. Specifically, the growth process was carried out at 710 °C for 180 min with the introduction of 350 and 30 sccm Ar and C_2_H_2_ gases simultaneously [46, 47]. After reaction, the CNCs were dispersed and washed by 68 wt% nitric acid and deionized (DI) water successively. The Fe_2_O_3_ and NiO nanoparticles were synthesized by using one-step hydrothermal method with the same reducing agent and reaction condition. Typically, 0.2 mmol soluble Fe^3+^ or Ni^2+^ salts were dissolved in 35 mL DI water, then 1.0 g urea was added into the mixture solution. After stirring vigorously for 30 min, the mixture was transferred into a 50 mL Teflon-lined autoclave and maintained at 160 °C for 6 h. The resulting precipitates were washed with DI water three times and then dried at 60 °C for 24 h. Figure S1 shows the SEM images of all as-obtained precursors.

### Preparation of RGO/CNC/M-NPs Aerogels

For the synthesis of RGO/CNC/M-NPs aerogels with different properties, A total of *X* (*X* = 0.2, 0.3) mmol of Fe_2_O_3_ and NiO mixed powders were added in 15 mL DI water, and the molar mass ratio of Fe_2_O_3_ and NiO was kept as 1:1. Afterward, 40 mg graphene oxide and 10 mg CNC powders were added to the mixture solution. After stirring and ultrasonication for 3 h, the solution was transferred into a 25 mL Teflon-lined autoclave and maintained at 180 °C for 12 h [48]. Subsequently, the resulting hydrogels were washed with deionized water and then freeze-dried at − 70 °C for 24 h. When the contents of added M-NPs were 0.2 and 0.3 mmol, the as-obtained samples were designated as GCA–M_0.2_–0 and GCA–M_0.3_–0, respectively. Meanwhile, additional control samples were also fabricated using 50 mg graphene oxide powder (without CNCs) in the same preparation condition, and the as-prepared samples were labeled as GA–M_0.2_–0 and GA–M_0.3_–0, respectively. Figure S2 shows the SEM images of all as-obtained aerogels.

### Preparation of RGO/CNC/CNF/M-NPs Aerogels

The RGO/CNC/CNF/M-NPs aerogels were synthesized by the CVD methods as follows. Prior to the process, 30 mg RGO/CNC/M-NPs aerogel was uniformly supported by the quartz boat. To prepare aerogels with different electromagnetic properties, the reaction time was controlled precisely. Specifically, the process was carried out at 450 °C with the introduction of 350 and 10 sccm Ar and C_2_H_2_ gases simultaneously. When the reaction time was 5, 10, and 20 min, the as-obtained samples were designated as GCA–M_0.2_–5, GCA–M_0.2_–10, GCA–M_0.2_–20, GCA–M_0.3_–5, GCA–M_0.3_–10, and GCA–M_0.3_–20, respectively.

### Characterization

The morphologies of samples were investigated by field-emission scanning electron microscope (FE-SEM, NOVA NanoSEM 450) operating at 3 kV, and transmission electron microscope (TEM) on a FEI Tecnai F30 operating at 300 kV. Meanwhile, the elemental mappings of the samples were also measured by energy dispersive X-ray spectrometers (EDX), which are equipped with the FE-SEM and TEM. The chemical composition of sample was identified by X-ray diffraction (XRD, PANalytical B.V diffractometer), X-ray photoelectron spectroscopy (XPS, VG ESCALAB 250Xi), and Raman spectroscopy (Renishaw in Via plus). The magnetic properties and surface areas of the samples were carried out by using a vibrating sample magnetometer (VSM, LakeShore-7300S) and a specific surface analyzer (QUADRASORB SI-KR/MP, Quantachrome, USA), respectively. The electrical conductivities of samples were measured using a classical 4-probe in-line contact method with a Keithley 2450 source/meter.

### Electromagnetic Measurements

To measure the complex electromagnetic parameters, as-obtained aerogels were crushed and mixed homogeneously with wax under the weight ratio of 15:85. Afterward, the mixture was pressed into a standard toroidal-shaped sample, with an inner diameter of 3.04 mm, outer diameter of 7 mm, and the thickness of 2 mm. Afterward, the network analyzer (Agilent 8720B) was used to measure the electromagnetic parameters in the range of 1–18 GHz at 293 K.

To evaluate the performance of the as-obtained EMAMs, the RL values in the frequency of 1–18 GHz were calculated on the basis of transmission line theory, which was depicted by Eqs. (1) and (2) [49–62]:

1 $${\text{RL}} = 20\,\log \left| {(Z_{{{\text{in}}}} - Z_{0} )/(Z_{{{\text{in}}}} + Z_{0} )} \right|$$

2 $$Z_{{{\text{in}}}} = Z_{0} \sqrt {\frac{{\mu_{{\text{r}}} }}{{\varepsilon_{{\text{r}}} }}} \tanh \left[ {\frac{{j\left( {2\pi fd} \right)}}{{c\sqrt {\varepsilon_{{\text{r}}} \mu_{{\text{r}}} } }}} \right]$$

where *Z*_0_ and *Z*_in_ are the impedance of free space and the normalized input impedance of absorber, respectively. Besides, the *f* and* c* represent the frequency of the microwave and the speed of light, respectively. Finally, *d* is the thickness of the absorber.

## Result and Discussion

### Morphology and Composition

**Fig. 2:**
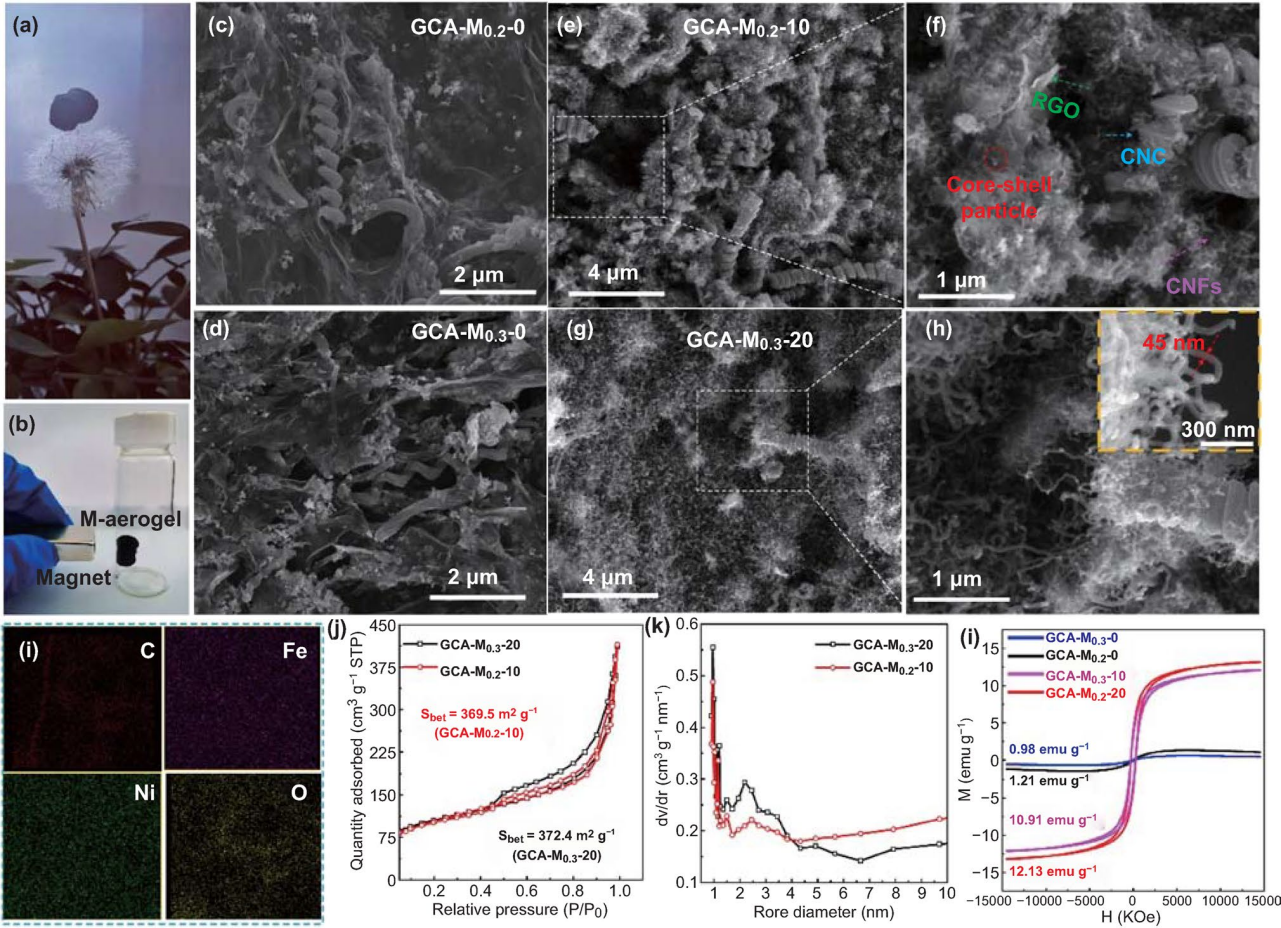
As-obtained aerogel (**a**) standing on a dandelion, and (**b**) attracting by a magnet; The SEM images of (**c**) GCA–M_0.2_–0, (**d**) GCA–M_0.3_–0, (**e**, **f**) GCA–M_0.2_–10, and (**g**, **h**) GCA–M_0.3_–20, (**i**) elemental mappings of C, Fe, Ni, and O in GCA–M_0.3_–20. (**j**) N_2_ adsorption/desorption isotherms and (**k**) corresponding pore size distributions for the GCA–M_0.2_–10 and GCA–M_0.3_–20. (**l**) Magnetic hysteresis loops of GCA–M_0.2_–0, GCA–M_0.3_–0, GCA–M_0.2_–10, and GCA–M_0.3_–20

The morphologies and typical properties of as-obtained aerogel were investigated. Figure 2a shows an optical image of a GCA–M_0.2_–10 aerogel supporting by a dandelion without any deformation of the stem, exhibiting the lightweight nature of the aerogel. Figures S3a and 2b show the optical image of a GCA–M_0.2_–0 aerogel before and after the CVD reaction, respectively. These results indicate that the magnetic aerogel was prepared after the CVD process. Furthermore, Fig. 2c, d display the morphologies of GCA–M_0.2_–0 and GCA–M_0.3_–0 aerogels, respectively. It is observed that the CNCs insert into the graphene layers, which boost the porosity of the samples. As a comparison, the RGO nanosheets in GA series samples (without CNCs) seriously stacked, and there are few porous structures in the samples (as indicated in Figs. S2a, b, e, and f). Meanwhile, by increasing the added content of M-NPs in the precursor, the nanoparticle density of GCA–M_0.3_–0 is obviously larger than that of GCA–M_0.2_–0. Figure 2e, f shows the SEM images of GCA–M_0.2_–10, it is found that abundant short 1D CNFs and 0D carbon particles emerge from the RGO/CNC skeleton. Figure 2f suggests the hierarchical aerogel comprising of 0-, 1-, 2-, and 3-D nano carbon cells maintains its porous structure well. With increasing the content of M-NPs and CVD reaction time, the aerogels with different morphologies were synthesized, as shown in Fig. 2g, h. It is observed that dense and long CNFs appear and almost fully cover the RGO/CNC skeleton. From the insert of Fig. 2h, it is seen that the typical 1D CNF with a fiber diameter of about 45 nm. A series of SEM analyses of the materials prepared under different conditions were conducted and given by Fig. S4. These results suggest that the growth density and length of CNFs increase with the increasing of the M-NPs content and CVD reaction time, respectively. The morphologies of the GA series aerogels after CVD reaction are observed by Fig. S5a, b. It is concluded that GCA series samples exhibit obvious porous structure than GA series samples. This result further proves that the introduction of CNC into the aerogel can increase porosity and give the necessary space for CNF growth. To explore the distributions of elements in GCA–M_0.3_–20, elemental distributions of carbon (C), iron (Fe), Nickel (Ni), and oxygen (O) were further investigated using EDX mapping. As shown in Fig. 2i, the Fe and Ni elements are well distributed throughout the carbon network. The nitrogen adsorption/desorption measurements were utilized to analyze the specific surface areas and pore structure of typical samples. As shown in Fig. 2j, the curves of GCA–M_0.2_–10 and GCA–M_0.3_–20 show an IV-type isotherm [63], and the long and narrow hysteresis loops at relative pressure from 0.45 to 1.0 are observed simultaneously. The calculated specific surface areas of GCA–M_0.2_–10 and GCA–M_0.3_–20 are 369.5 and 372.4 cm^2^ g^−1^, respectively. Besides, the pore distribution properties of the as-mentioned samples are given by Fig. 2k. It is confirmed that a nano- and meso-porous hybrid matrix exists in the samples [64]. Furthermore, as shown in Fig. S5e, the calculated specific surface areas of GA–M_0.2_–10 and GA–M_0.3_–20 are only 245.4 and 257.1 cm^2^ g^−1^, respectively. The above results well prove that the introduction of CNC would increase the porosity of aerogel. Meanwhile, a comparison table of specific surface area for RGO-based hierarchical materials is given in Table S1. According to the comparison table, it is concluded that the use of CNC to decrease graphene stacking and agglomeration is an effective strategy. Furthermore, magnetic hysteresis loops of typical samples are given by Fig. 2l, and the saturation magnetization values for GCA–M_0.2_–0, GCA–M_0.3_–0, GCA–M_0.2_–10, and GCA–M_0.3_–20 are 0.98, 1.21, 10.91, and 12.13 emu g^−1^, respectively. These results further certify that the non-magnetic sample is converted to a magnetic one after the CVD process, which endows as-obtained aerogel with the magnetic loss.

**Fig. 3:**
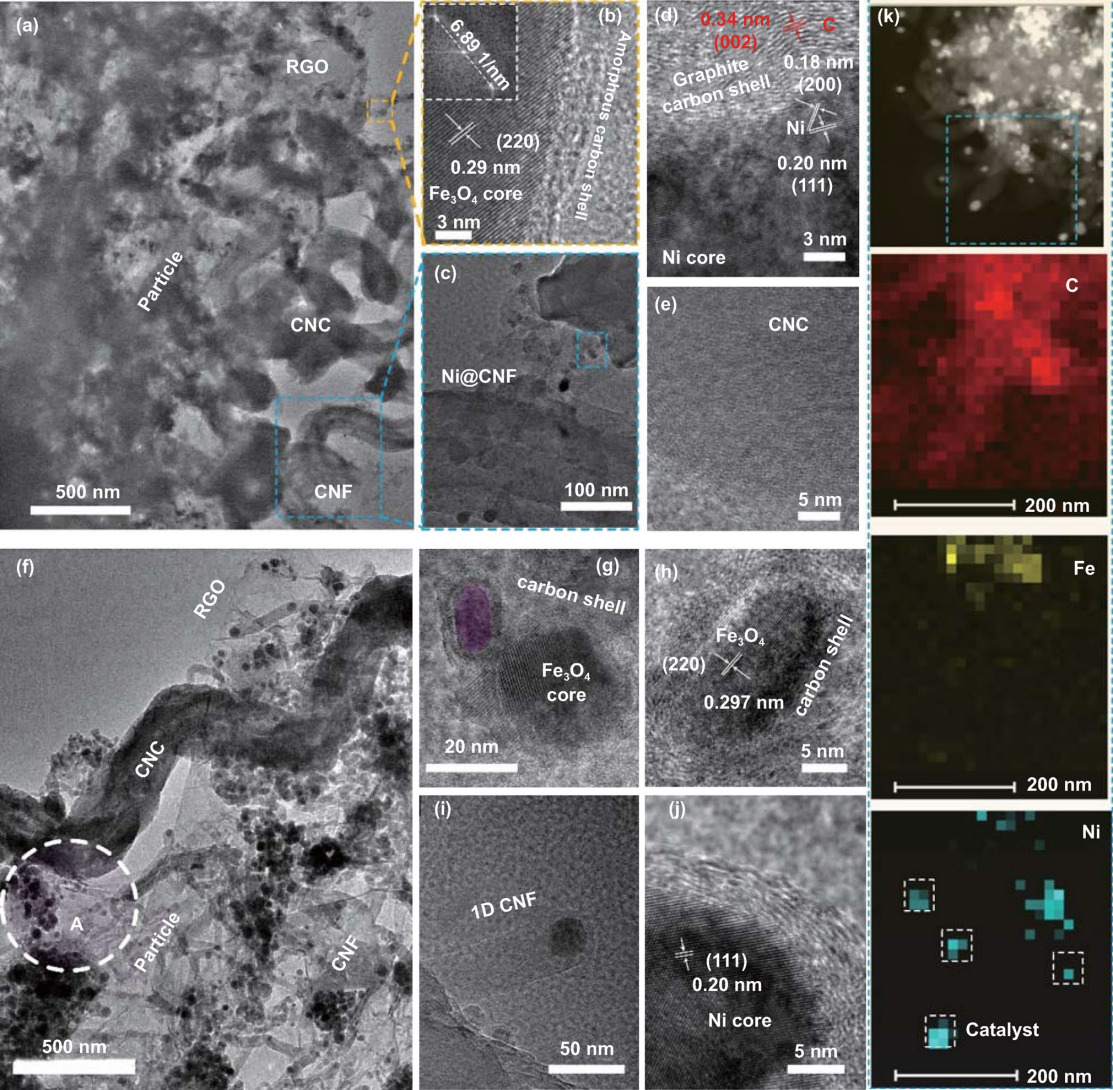
(**a**) TEM image of GCA–M_0.2_–10. Corresponding HRTEM images of (**b**) Fe_3_O_4_@C (insert of **b** FFT image), (**c**, **d**) carbon nanofiber, and (**e**) CNC. (**f**) TEM image of GCA–M_0.3_–20. Corresponding HRTEM images of (**g**, **h**) Fe_3_O_4_@C and (**i**, **j**) carbon nanofiber. (**k**) Elemental maps of C, Fe, and Ni in the GCA–M_0.3_–20 sample

The morphologies and internal structures of typical samples were systematically observed by TEM and HRTEM images. It is clearly seen that the GCA–M_0.2_–0 aerogel consists of graphene nanosheets, helical CNCs, and M-NPs (as shown in Fig. S6a). Meanwhile, it is certified that no CNFs or carbon shell (Fig. S6b) are synthesized before the CVD process. As displayed in Fig. 3a, the GCA–M_0.2_–10 aerogel with hierarchical and porous structure appeared, and some CNFs and nanoparticles are synthesized and deposited on the skeleton of RGO/CNC. The HRTEM images further confirm the internal structures of 0D core-shell nanoparticles and 1D CNFs. In Fig. 3b, the HRTEM and its corresponding FFT images confirm that the interplanar spacing of 0.29 nm can correspond to the (220) plane of Fe_3_O_4_ [65]. Meanwhile, the thin layer of amorphous carbon covers the Fe_3_O_4_ core, which convincingly confirms the formation of core-shell structured Fe_3_O_4_@C. Furthermore, as shown in Fig. 3c, the catalyst Ni particles are encapsulated on the top of short and irregular CNFs. Besides, three crystal interplanar spacings (Fig. 3d), 0.18, 0.20, and 0.34 nm, could be well fitted with the (200), (111) of Ni, and (002) of graphitic carbon, respectively [66].These results suggest that the Fe_2_O_3_ and NiO are partially reduced to Fe_3_O_4_ and Ni during the CVD process, which play the role in catalytically synthesizing the carbon shell and CNFs, respectively. Furthermore, the HRTEM image of CNC was given by Fig. 3e, it is observed that the crystallinity of the CNC is poor because the orientation of crystallites is disordered. For comparison, the TEM and HRTEM images of GCA–M_0.3_–20 were given by Fig. 3f–j. As shown in Fig. 3f, the 3D CNC, 2D RGO, 1D CNFs, and 0D M-NPs are obviously observed. With the increase in the reaction time, the Fe_3_O_4_@C nanoparticle with a thicker carbon shell is seen in Fig. 3g, h. The area marked A in Fig. 3f is carefully observed. A large number of particles with a core-shell structure were confirmed in Fig. S7. Besides, the internal particle with a lattice spacing of 0.297 nm is well in line with the (220) plane of Fe_3_O_4_ (Fig. 3h). The HRTEM image of the catalyst particle is given by Fig. 3j, the typical lattice interlayer with the distance of 0.20 nm correspond to the (111) crystal plane of Ni [67]. Besides, to further elucidate the effects of Fe_2_O_3_ and NiO in the precursor, the EDX mapping images are used to investigate the element distributions of GCA–M_0.3_–20. As shown in Fig. 3k, the distributions of Ni and Fe are obviously different. More importantly, when we check the tip area of the CNFs, only the signal of Ni appears, which strongly suggests that the NiO particles play the role of the catalysts for CNF growth (as indicated in Fig. S8). Clearly, based on the above results of morphology and composition, hierarchical architecture with “helix-sheet–fiber–dot” structure is successfully synthesized.

**Fig. 4:**
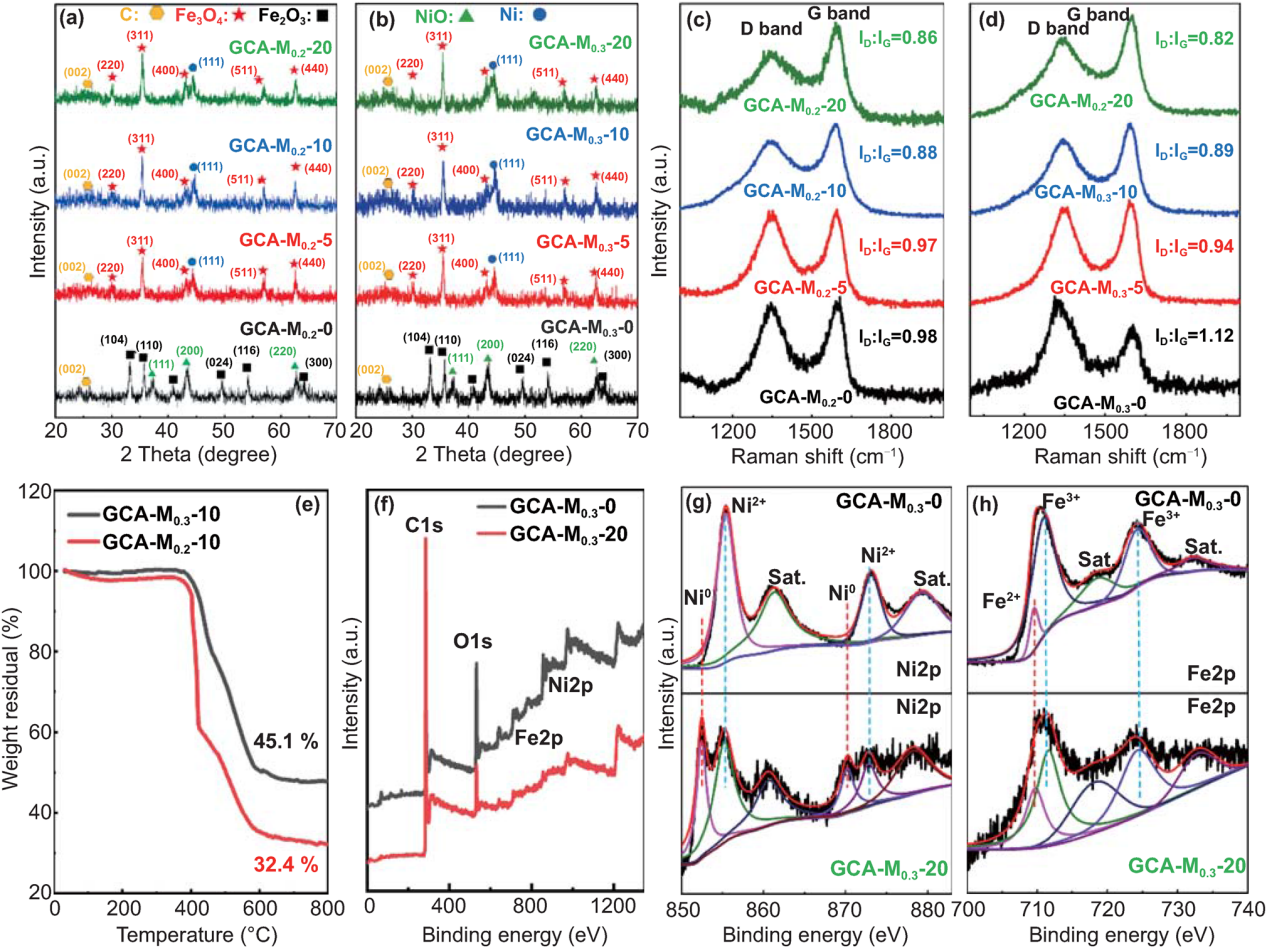
(**a**, **b**) XRD patterns of GCA–M_0.2_–*Y* and GCA–M_0.3_–*Y*, respectively. (**c**, **d**) Raman spectra of GCA–M_0.2_–*Y* and GCA–M_0.3_–*Y*, respectively. (**e**) Thermogravimetric curves of GCA–M_0.3_–10 and GCA–M_0.2_–10. (**f**) XPS survey spectra of GCA–M_0.3_–0 and GCA–M_0.3_–20. (**g**, **h**) High resolution XPS spectra of Ni 2*p* and Fe 2*p* for GCA–M_0.2_–Y and GCA–M_0.3_–Y

The crystal and phase structure of the GCA–M_0.2_–*Y* and GCA–M_0.2_–*Y* (*Y* = 0, 5, 10, and 20) aerogels were characterized by XRD patterns and Raman spectra. Figure 4a, b shows the XRD patterns of the samples. It is observed that the diffraction peaks of GCA–M_0.2_–0 and GCA–M_0.3_–0 are in accordance with standard records of α-Fe_2_O_3_ and NiO (JCPDF No. 33-0664 and 89-7130) [68, 69]. After the CVD process, the XRD diffraction peaks of GCA–M_0.2_–*Y* and GCA–M_0.3_–*Y* shift obviously. The typical diffraction peaks at 18.3°, 30.6°, 35.5°, 43.0°, 57.2°, and 62.7° correspond well to the (220), (311), (400), (511), and (440) planes of Fe_3_O_4_ (JCPDF No. 19-0629). Meanwhile, the typical peak at 44.8° corresponding to the (111) plane would be assigned to face-centered cubic Ni (JCPDF No. 04-0850 [7, 65]. Meanwhile, the wide peaks at about 25° in the XRD patterns are ascribed to the existence of the carbon materials (JCPDF No. 50-0926). On the other hand, The Raman spectrometer was further employed to evaluate the carbon qualities. Figure 4c, d exhibits the Raman spectrums of the GCA-M_0.2_–*Y* and GCA–M_0.3_–*Y* aerogels, respectively. The comprehensive information is investigated by the intensity ratio of the D and G peaks of the carbon (*I*_D_/*I*_G_). It is concluded that with the continuous increase in CVD reaction time, the values of *I*_D_/*I*_G_ decrease, which may be due to the density and length of graphited CNFs increases. This result indicates that the conductivity and conductive loss of samples could be orderly tailored by introducing 1D CNFs. Meanwhile, carbon content changes of GCA–M_0.3_–10 and GCA–M_0.2_–10 were investigated using thermogravimetric analysis (TGA). As shown in Fig. 4e, the residual contents of GCA–M_0.3_–10 and GCA–M_0.2_–10 reach to 45.1 and 32.4 wt%, respectively. This result proves that the content of carbon decreases by increasing the content of metal oxide particles. On the other hand, to clarify the changes of chemical valence states before and after the CVD process, the samples were examined by the XPS technique. As shown in Fig. 4f, the XPS full-survey-scan spectrums of GCA–M_0.3_–0 and GCA–M_0.3_–20 confirm the presence of C, O, Fe, and Ni elements. Furthermore, for GCA–M_0.3_–0, the high-resolution XPS spectrum of the Ni displays two characteristic peaks at 856.2 and 873.7 eV, which are assigned to Ni 2*p*_1/2_ and 2*p*_3/2_ peaks of Ni^2+^, respectively (Fig. 4g). However, for GCA–M_0.3_–20, the characteristic peaks centering at 853.0 and 870.2 eV are assigned to the form of Ni^0^, and the rest of the peaks corresponded to the position of Ni^2+^ [70]. Besides, as shown in Fig. 4h, the Fe 2*p* spectrum for GCA–M_0.3_–0 mainly consists of a series of characteristic peaks of Fe^3+^, and the corresponding Fe 2*p*_3/2_ peak at 711.2 eV and the Fe 2*p*_1/2_ peak at 725.1 eV are in agreement with previously reported data [65]. For GCA–M_0.3_–20, the intensity of characteristic peak for Fe^2+^ at 710.1 eV is significantly enhanced, which is ascribed to the formation of Fe_3_O_4_ [71]. The XPS results further confirm that the Fe_2_O_3_ and NiO in precursor are reduced to Fe_3_O_4_ and Ni, respectively.

### Microwave Absorption Performance of As-obtained Aerogels

**Fig. 5:**
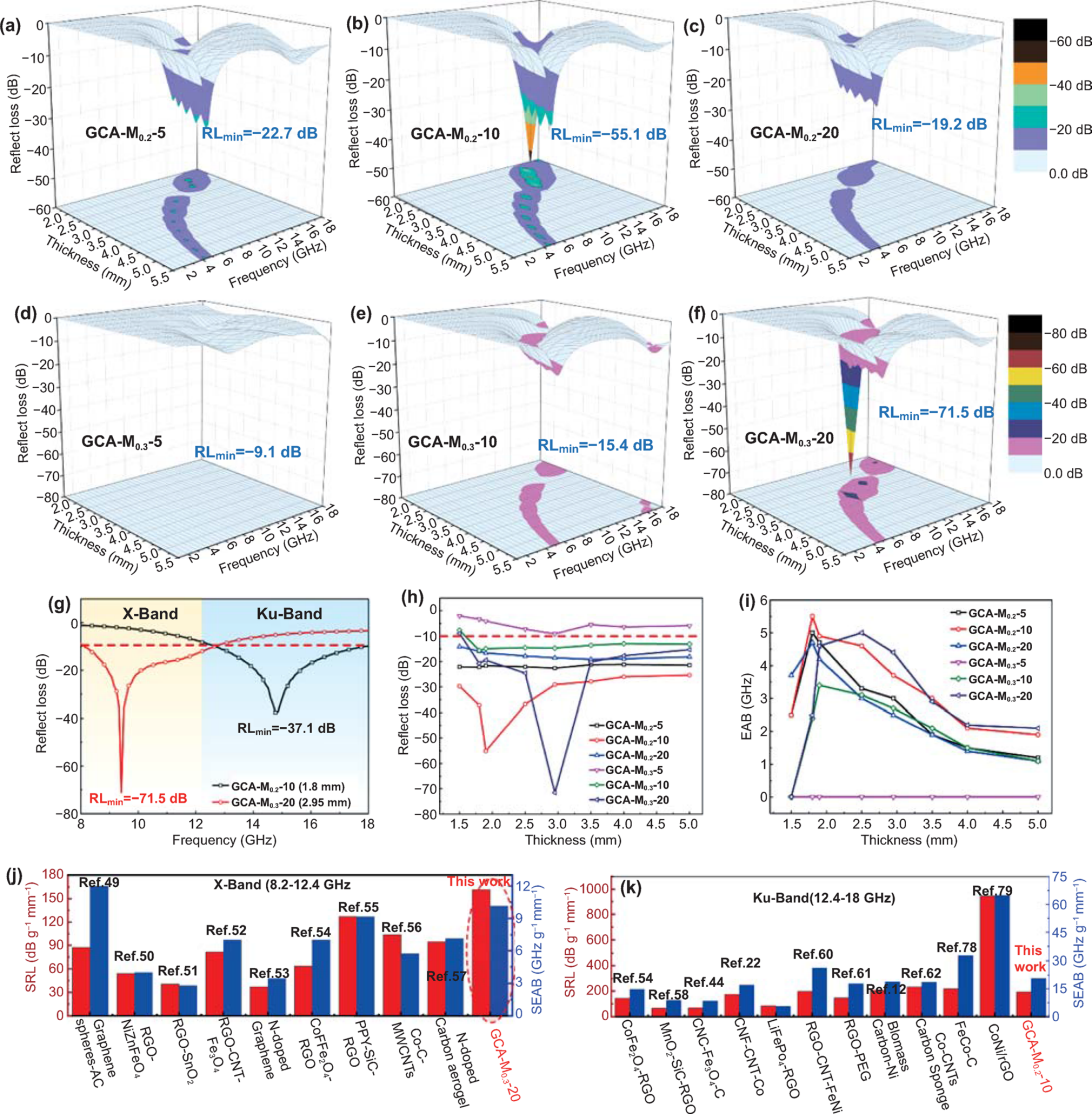
3D representations of the RL of (**a**) GCA–M_0.2_–5, (**b**) GCA–M_0.2_–10, (**c**) GCA–M_0.2_–20, (**d**) GCA–M_0.3_–5, (**e**) GCA–M_0.3_–10, and (**f**) GCA–M_0.3_–20. (**g**) Maximum EABs of the GCA–M_0.2_–10 and GCA–M_0.3_–10 aerogels. (**h**, **i**) RL_min_ and EAB values of as-obtained samples with different thicknesses, respectively. (**j**, **k**) Comparison of specific RL and specific EAB values of recently reported carbon-based aerogels in X- and Ku-band

Figure 5a–f gives the 3D RL curves of the as-obtained composites at different thicknesses with only 15% filling ratio. In general, the performances of EMAMs are evaluated using the optimal value of RL_min_ and EAB. As shown in Fig. 5a and S9a, the RL_min_ value of GCA-M_0.2_–5 is − 22.7 dB at the thickness of 5.5 mm. However, the smaller part of the EAB region suggests poor performance. Furthermore, the RL_min_ value of GCA–M_0.2_–10 arrives − 55.1 dB at 13.8 GHz, and the corresponding EAB is 5 GHz with a thickness of only 1.9 mm. Moreover, when the thickness of absorber reduces to 1.8 mm, the value of EAB is up to 5.6 GHz from 12.4 to 18 GHz, almost covering the 93% of Ku band (Fig. 5b, g and S9b). Besides, as displayed by Fig. 5c and S9c, the GCA–M_0.2_–20 exhibits a degraded RL_min_ value of only − 19.2 dB, and the corresponding EAB exceeding − 10 dB is 4.8 GHz. Moreover, the microwave attenuation capacity of samples would also be adjusted by tailoring the content of M-NPs. Unfortunately, with the increase in M-NPs content, the GCA–M_0.3_–5 and GCA–M_0.3_–10 exhibit the negligible values of RL_min_ and EAB (Fig. 5d, e and S9d, e). Surprisingly, for sample GCA–M_0.3_–20 (Fig. 5f and S9f), it exhibits the outstanding *RL*_min_ value of − 71.5 dB at 9.5 GHz with a thickness of 2.95 mm. Moreover, the broaden EAB of 4.5 GHz (8.2–12.7 GHz) is obtained, which covers the whole X band (Fig. 5g). In addition, the microwave absorbing performance of the samples before the CVD reaction (GCA–M_0.2_–0 and GCA–M_0.3_–0) has also been tested. Due to the weak dielectric loss and magnetic loss, their exhibit almost negligible performance (as indicated in Fig. S10). Figure 5h, i compares, in detail, the microwave absorption performances of as-obtained GCA–M_*X*_–*Y* (*X* = 0.2, 0.3, and *Y* = 5, 10, 20, respectively) with thicknesses of 1.5–5.0 mm. Clearly, it is observed that GCA–M_0.2_–10 and GCA–M_0.3_–20 exhibit outstanding performance at the relatively thin thickness. Furthermore, we compared the microwave absorption performances of GCA–M_0.2_–10 and GCA–M_0.3_–20 with the state-of-the-art carbon-based absorbers (published during the last 3 years). For the comprehensive comparison regarding performances of the as-mentioned EMAMs, the specific RL (SRL, RL_min_/(thickness × filler loading)) and specific EAB (SEAB, EAB/(thickness × filler loading)) values are calculated and given by Fig. 5j and k, respectively. Among all, The GCA-M_0.3_–20 fabricated in this work exhibits a superior SRL value (161.5 dB mm^−1^ g^−1^) in X-band, and the SEAB value of GCA–M_0.3_–20 (10.16 GHz mm^−1^ g^−1^) is also relative superior to other listed EMAMs (Fig. 5j). Besides, it is clear from Fig. 5k that the SRL and SEAB values of GCA–M_0.2_–10 exhibit advantages by overall consideration of absorber loading (< 20 wt%) and thickness (< 2 mm) in Ku-band. On the other hand, the corresponding tables (Tables S2 and S3) including each works from the literatures, not only the RL value, but also the frequency range, the typical thickness, and the filling ratio of the samples are summarized in supplementary materials. According to the above comparison, it is concluded that the as-obtained hierarchical aerogels in this paper would act as an extremely promising candidate for a lightweight, thin, and highly efficient MWAMs.

### Electromagnetic Parameters of As-obtained Aerogels

**Fig. 6:**
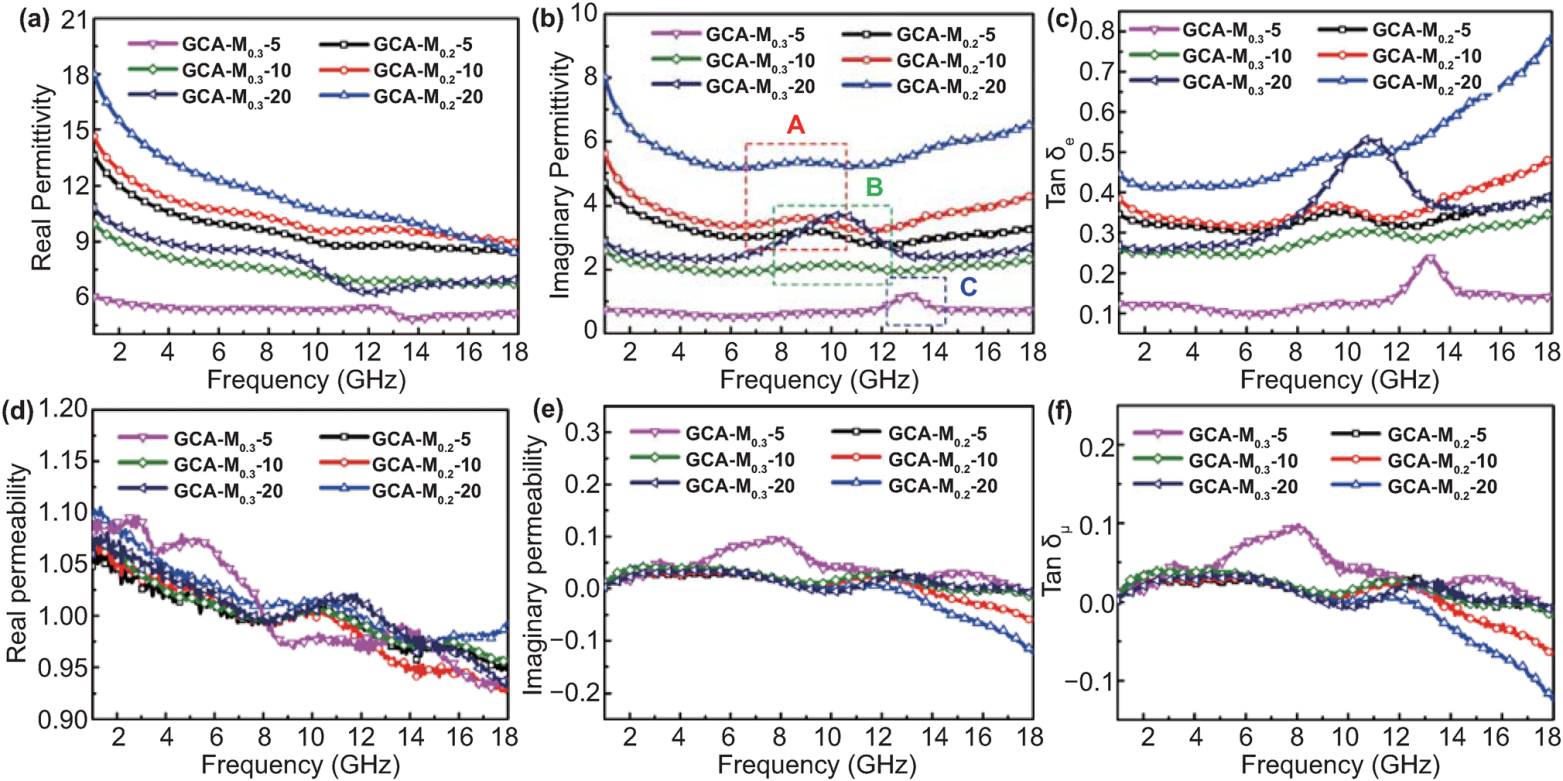
Frequency dependence of (**a**) real permittivity, (**b**) imaginary permittivity, (**c**) dielectric loss tangent, (**d**) real permeability, (**e**) imaginary permeability, and (**f**) magnetic loss tangent

The dependence of the complex permittivity ($\varepsilon_{{\text{r}}} = \varepsilon^{\prime}_{{\text{r}}} - \varepsilon^{\prime\prime}_{{\text{r}}}$) and complex permeability ($\mu_{{\text{r}}} = \mu^{\prime}_{{\text{r}}} - \mu^{\prime\prime}_{{\text{r}}}$) on frequency from 1 to 18 GHz was further discussed for understanding the microwave absorbing mechanism of as-obtained aerogels, and the results were given by Fig. 6. All in all, the real parts ($\varepsilon^{\prime}_{{\text{r}}}$ and $\mu^{\prime}_{{\text{r}}}$) of the electromagnetic parameters represent the storage capability of electromagnetic wave energy, and the imaginary parts ($\varepsilon^{\prime\prime}_{{\text{r}}}$ and $\mu^{\prime\prime}_{{\text{r}}}$) stand for the ability of energy dissipation [9]. It is observed that all the $\varepsilon^{\prime}_{{\text{r}}}$ values exhibit a decreasing tendency in the frequency range of 1 to12 GHz, then display the relative constant values in 12–18 GHz (Fig. 6a). Meanwhile, as shown in Fig. 6b, three remarkable vibration peaks are observed at ~ 8.8 GHz (labeled as A), ~ 10.5 GHz (labeled as B), and ~ 13.2 GHz (labeled as C), which suggest relaxation behavior exist in $\varepsilon^{\prime\prime}_{{\text{r}}}$. Considering the dimensional gradient of as-prepared samples, it is reasonable that the local space charge accumulation of heterojunction interface and the surface geometry induce enhancement effect for dielectric loss [16]. Besides, the dielectric loss tangent tanδ_e_ values were computed to evaluate dielectric dissipation capability. The order of tanδ_e_ values for GCA–M_*X*_–*Y* samples is GCA–M_*X*_–20 > GCA–M_*X*_–10 > GCA–M_*X*_–5 (*X* = 0.2, 0.3), which gives the evidence that the introduction of 1D CNFs is an effective strategy to boost the dielectric attenuation capacity. Meanwhile, it should be noted that the conductive paths composed of the 1D structures are the key to improving the dielectric loss. To solidate this viewpoint, the electromagnetic parameters of GCA–M_0.2_–0 and GCA–M_0.3_–0 were tested and compared with those of GCA–M_0.2_–10 and GCA–M_0.3_–20, respectively. After the introduction of CNFs (as shown in Fig. S11a, b), the imaginary part of complex permittivity is significantly increased, which suggests the improvement of dielectric loss (as indicated by Fig. S11c, d). Moreover, the dielectric loss mechanism is analyzed by the relationship between $\varepsilon^{\prime}_{{\text{r}}}$ and $\varepsilon^{\prime\prime}_{{\text{r}}}$. Based on the Debye relaxation theory, if the curve of $\varepsilon^{\prime}_{{\text{r}}}$ versus $\varepsilon^{\prime\prime}_{{\text{r}}}$ exhibits semicircle morphology, these MWAMs would possess strong polarization behavior, and each semicircle is denoted as Cole–Cole semicircle [29]. For GCA–M_0.2_–*Y* samples, except for the two semicircles, the corresponding curves also possess a distinguishable straight line in the tail, suggesting that the high conductive loss originating from the conductive 3D carbon network in the MWAMs/wax composites (Fig. S12a–c). Meanwhile, GCA–M_0.3_–*Y* samples exhibit three Cole–Cole semicircles and shortened “line tail”, indicating the enhanced polarization relaxation and relatively low conductive loss [2]. These results mentioned above reveal that the dielectric loss of GCA-M_X_-Y originates approximatively from relaxation loss and conductive loss. Besides, Fig. 6d–f reveal the complex permeability of the as-prepared aerogels. Briefly, the real ($\mu^{\prime}_{{\text{r}}}$) and imaginary ($\mu^{\prime\prime}_{{\text{r}}}$) parts of all the samples are almost near 1 and 0, respectively, suggesting the magnetic loss is relatively weak due to the inconspicuous natural magnetic resonance (Fig. 6e) and eddy current loss (Fig. S13a). Meanwhile, the negligible tanδ values of as-obtained aerogels also prove this viewpoint (Fig. 6f). Even so, the presence of magnetic loss would ameliorate the impedance match and boost the dissipation of electromagnetic energy. Moreover, the manipulated $\mu^{\prime}_{{\text{r}}}$ and $\mu^{\prime\prime}_{{\text{r}}}$ values could induce the absorption peaks to transfer to a lower frequency.

In general, the ideal MWAMs should possess powerful electromagnetic wave dissipation ability and excellent impedance matching synchronously. Normally the attenuation constant (*α*) is used for the analysis of the inherent attenuation ability and calculated by Eq. (3) [44]:

3 $$\alpha = \frac{\sqrt 2 \pi f}{c}\sqrt {\left( {\mu^{\prime\prime}_{{\text{r}}} \varepsilon^{\prime\prime}_{{\text{r}}} - \mu^{\prime\prime}_{{\text{r}}} \varepsilon^{\prime\prime}_{{\text{r}}} } \right) + \sqrt {\left( {\mu^{\prime\prime}_{{\text{r}}} \varepsilon^{\prime\prime}_{{\text{r}}} - \mu^{\prime\prime}_{{\text{r}}} \varepsilon^{\prime\prime}_{{\text{r}}} } \right)^{2} + \left( {\mu^{\prime\prime}_{{\text{r}}} \varepsilon^{\prime\prime}_{{\text{r}}} + \mu^{\prime\prime}_{{\text{r}}} \varepsilon^{\prime\prime}_{{\text{r}}} } \right)^{2} } }$$

**Fig. 7:**
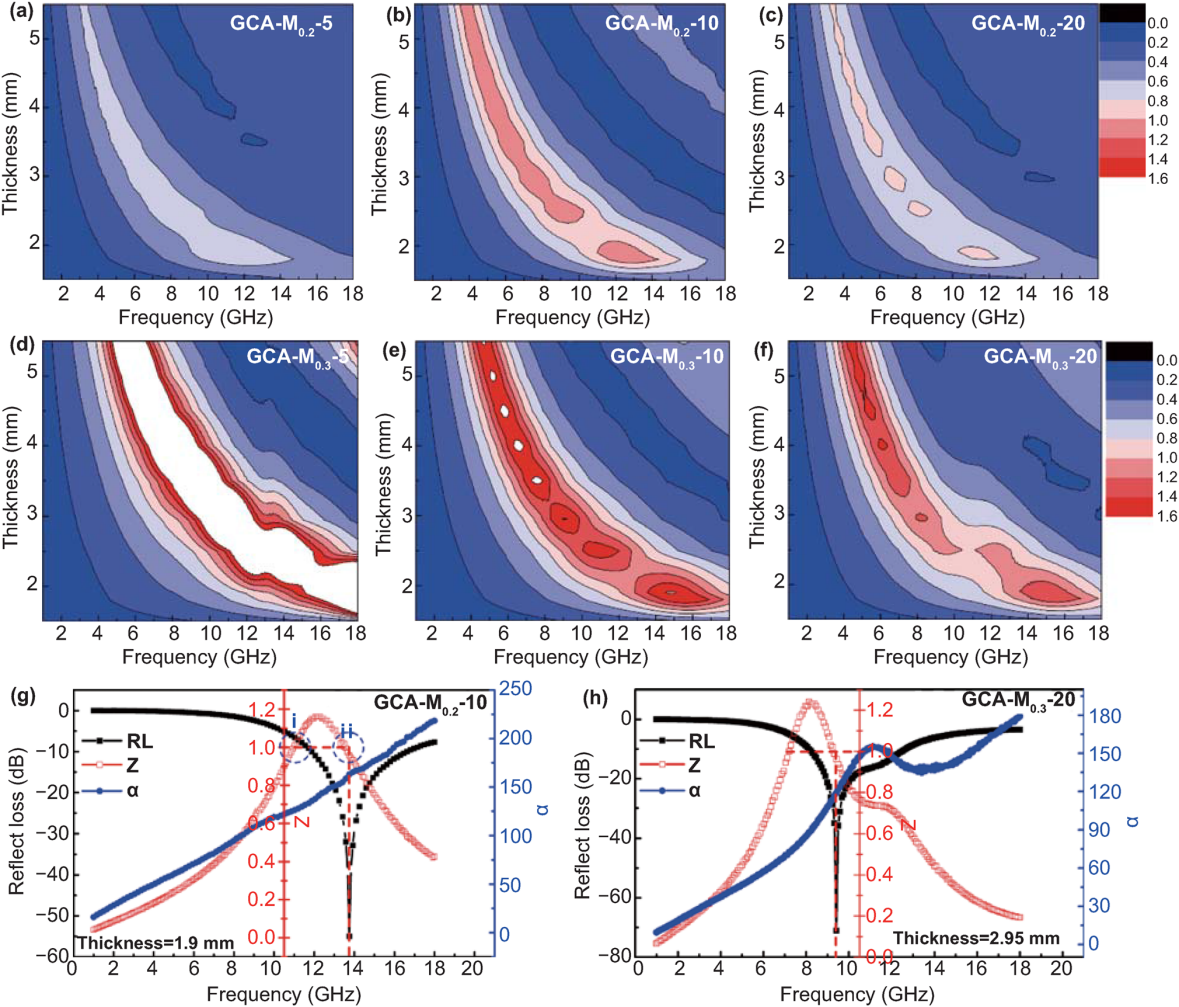
Calculated *Z* values at different thicknesses from 1 to 18 GHz for (**a**) GCA–M_0.2_–5, (**b**) GCA–M_0.2_–10, (**c**) GCA–M_0.2_–20, (**d**) GCA–M_0.3_–5, (**e**) GCA–M_0.3_–10, and (**f**) GCA–M_0.3_–20. Frequency-dependent RL, *α*, and *Z* values for (**g**) GCA–M_0.2_–10 and (**h**) GCA–M_0.3_–20

It is observed that all the *α* values increase with elevating the frequency and approximatively decrease with increasing M-NPs content (Fig. S13b). Furthermore, GCA–M_0.2_–20 shows the largest α value in the whole test frequency range. However, owing to the superfluous dielectric loss of GCA–M_0.2_–20, seriously impedance mismatching occurs and results in a poor microwave absorption performance. Therefore, the impedance matching $({\text{Z}}=\left|{\text{Z}}_{\text{in}}/{\text{Z}}_{0}\right|$) performance must be taken into account of microwave absorption process [72–77]. Based on previous research, when the *Z* value is equal or close to 1, the incident electromagnetic wave would easily enter the MWAM and then be converted to other forms of energy. The *Z* values of GCA–M_*X*_–*Y* aerogels at different thicknesses are computed and displayed in Fig. 7a–f. It can be seen that GCA–M_0.2_–10 has good impedance matching performance in almost the entire frequency range, which is derived from appropriate conductive loss and corresponding magnetic loss [78–81]. As a comparison, the *Z* value of the GCA–M_0.3_–5 is far from 1, and most of them are over 1.6 in the whole frequency range, suggesting the poorest impedance matching performance among all the samples. Linking back to the poor tan*δ*_e_ and non-negligible tan*δ*_e_ values for GCA–M_0.3_–5, it is reasonable that the matched dielectric and magnetic loss are two prerequisites for as-prepared aerogels to achieve the outstanding impedance matching performance. Furthermore, take GCA–M_0.2_–10 and GCA–M_0.3_–20 as the examples, the relationship between the RL_min_, *α*, and *Z* values is analyzed and given by Fig. 7g, f, respectively. It is clearly seen that as *Z* value is equal to 1, the RL values arrived at the minimum at 13.8 and 9.5 GHz for GCA–M_0.2_–10 and GCA–M_0.3_–20, respectively. Furthermore, we select GCA–M_0.2_–10 as an illustration to analyze the effort of *α* and *Z* on RL value. As shown in Fig. 7g, two points reach 1 in the *Z* curve, which are noted as *i* and *ii*. It is seen that point *ii* exhibits better microwave absorption performance than that of point *i*, suggesting larger *α* would boost RL_min_ value under the same *Z* value. All these results discussed above certify that the stronger attenuation ability with excellent impedance matching is a key point for our aerogel as an ideal MWAM.

### Roles of CNC

**Fig. 8:**
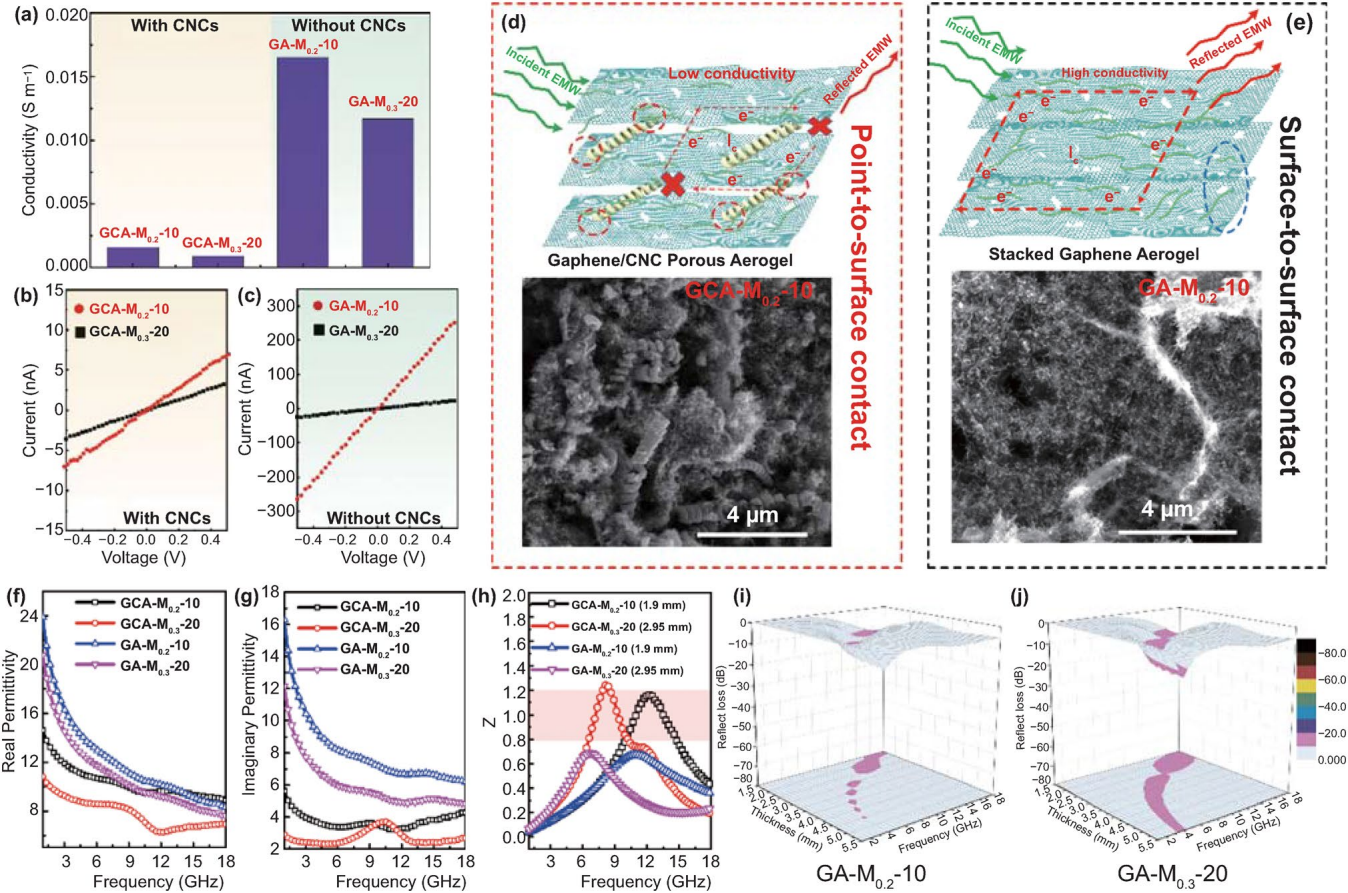
(**a**) Electrical conductivities of GCA–M_0.2_–10-, GCA–M_0.3_–20-, GA–M_0.2_–10-, and GCA–M_0.3_–20-wax composites. (**b**) Measured *I–V* curves of GCA–M_0.2_–10- and GCA–M_0.3_–10-wax composites. (**c**) Measured *I–V* curves of GA–M_0.2_–10- and GA–M_0.3_–20-wax composites. (**d**, **e**) Schematic of structure–function effects of CNC on dielectric property of as-obtained aerogels. (**f**) Real permittivity, (**g**) imaginary permittivity, and (**h**) *Z* values for GCA–M_0.2_–10-, GCA–M_0.3_–20-, GA–M_0.2_–10-, and GA–M_0.3_–20-wax composites

In order to clarify the key functions of the CNC in the system, we prepared two control aerogels without CNC, and the corresponding samples were labeled as GA–M_0.2_–10 (Fig. S5a) and GA–M_0.3_–20 (Fig. S5b), respectively. Typically, the dielectric performance of MWAMs is highly related to their electrical conductivity, and Fig. 8a gives the measured conductivities of as-prepared aerogel/wax composites. The electrical conductivities of GCA–M_0.2_–10-, GCA–M_0.3_–20-, GA–M_0.2_–10-, and GCA–M_0.3_–20-wax are 1.58 × 10^–3^, 0.89 × 10^–3^, 1.65 × 10^–2^, and 1.17 × 10^–2^ S m^−1^, respectively. It is concluded that the electrical conductivity obviously degrades with adding the CNCs in aerogel, and the measured *I*–*V* curves (Fig. 8b, c) are also consistent with this viewpoint. At present, most researchists have agreed that the conductivity of the sample is closely related to the crystal structure and morphology of materials [82–86]. In this case, the crystal structure of CNC is almost amorphous (Fig. 3e), which indicates the conductivity of the CNC lower than that of graphene and CNF. More importantly, as shown in Figs. 8d, e, and S14, the helical CNC are inserted graphene layers, resulting in the formation of point-to-surface contact (CNC-graphene) instead of surface-to-surface contact (graphene–graphene). Therefore, the evolution from surface-to-surface contact to point-to-surface contact leads to a decrease in electrical conductivity. Besides, the existence of helical CNCs would create the gaps and pores between the graphene layers, which lengthen the transmission distances of microwave owing to the multiple reflect and scatter effects. Moreover, based on the Maxwell–Garnett theory, the abundant void spaces of as-obtained hierarchical aerogels would effectively reduce the complex permittivity, thus improving impedance matching [52]. As a result, the dielectric property would change dramatically. To further shed light on the effect of the CNC skeleton in an aerogel, electromagnetic parameters of the GA–M_0.2_–10 and GA–M_0.3_–20 were measured and compared, as shown in Fig. 8f, g, and S10. For a comparison purpose, we also give the electromagnetic parameters of the GCA–M_0.2_–10 and GCA–M_0.3_–20. As shown in Fig. S15, the complex permeability of the GCA–M_*X*_–*Y* aerogels is similar to that of GA–M_*X*_–*Y* aerogels. This result suggests that the effect of CNCs on permeability is negligible. Besides, the $\varepsilon^{\prime}_{{\text{r}}}$ and $\varepsilon^{\prime\prime}_{{\text{r}}}$ values of GA–M_*X*_–*Y* samples are obviously higher than those of GCA–M_*X*_–Y samples, suggesting the aerogels without CNCs possess better dielectric dissipation capability in the test frequency range. Furthermore, the attenuation constants of typical GA–M_*X*_–*Y* and GCA–M_*X*_–*Y* samples are shown in Fig. S16. It is noted that the *α* of GA–M_*X*_–*Y* is higher than those of other GCA–M_*X*_–*Y* samples in the almost whole frequency range, which could be owed to the significantly enhanced conductive loss. However, the impedance matching of GCA–M_*X*_–*Y* aerogel seems to be much better than that of GA–M_*X*_–*Y* sample, which further proves the crucial role of CNC. As a result, the GA–M_*X*_–*Y* samples exhibit the poor performances of RL_min_ and EAB (Fig. 8i, j). Based on the above results, accounting for the effects arising from the amorphous structure of CNC, point-to-surface contact between CNC and graphene, and the concomitant porosity, the CNC-contained aerogels eventually exhibited a significantly improved impedance matching and microwave absorption performance.

### Microwave Absorption Mechanism

**Fig. 9:**
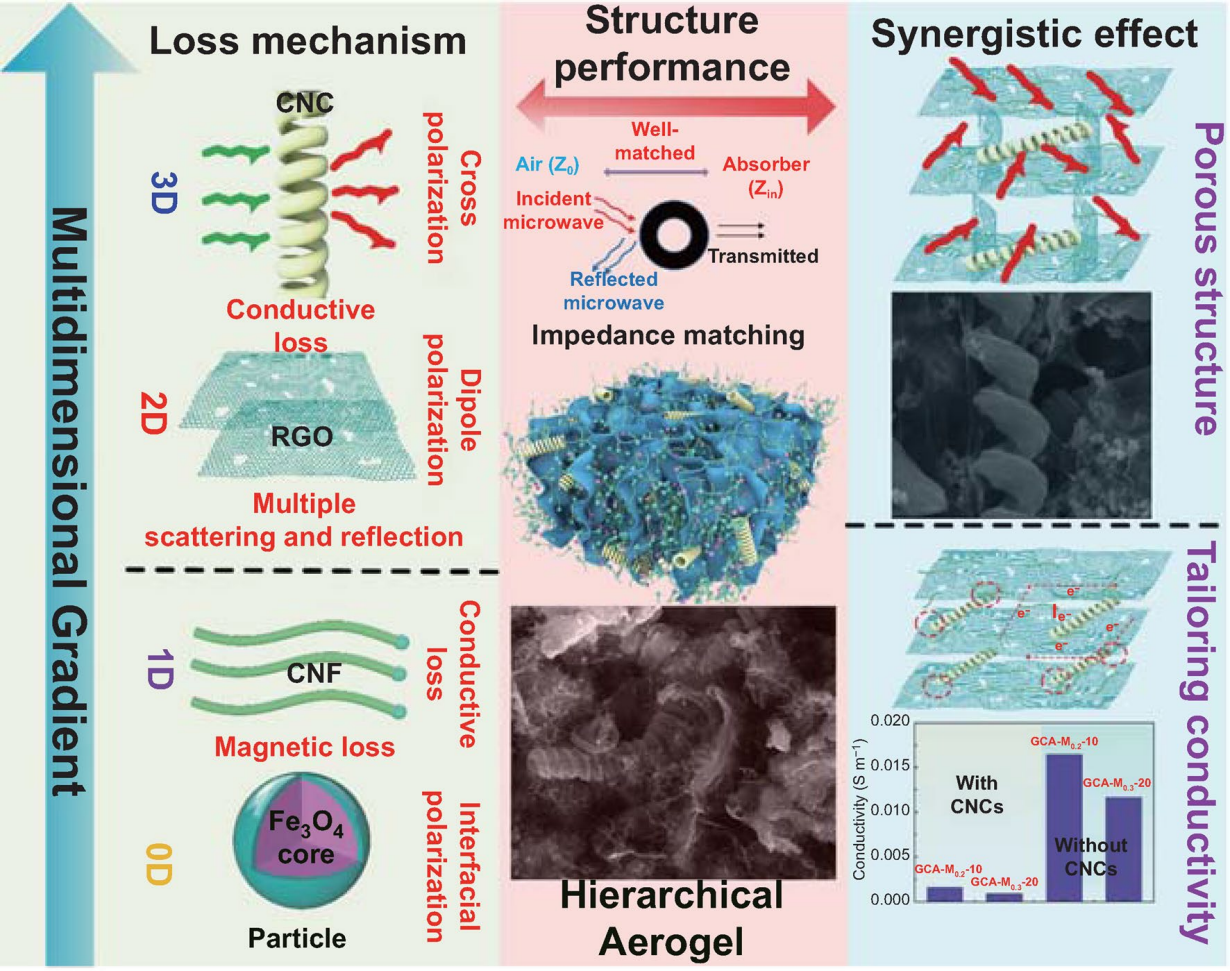
Pictorial description of relative microwave loss mechanisms existing in the GCA–M_*X*_–*Y*-wax composites

The superior microwave absorption performance of the as-prepared aerogel is closely related to the multiple loss mechanisms of multi-dimensional gradient structures and the synergistic effects of these components. In view of these facts, the microwave absorption mechanism is graphically summarized in Fig. 9. Firstly, the detailed loss mechanisms of these components in aerogels are analyzed as following. In general, there are six factors that dominate the microwave attenuation, including conductive loss, cross-polarization, dipole polarization, multiple scattering and reflection, interfacial polarization, and magnetic loss.

**For 3D structured CNC:** On the one hand, CNCs would induce cross-polarization loss due to their chiral structures during the interaction with electromagnetic wave. Besides, as one of the framework materials, conductive CNCs play an important role in the formation of the conductive network in the aerogel.

**For 2D structured RGO:** When the electromagnetic wave transmits into the absorber, it would be repeatedly scattered by the RGO nanosheets and thus boosts the multiple scattering loss. On the other hand, the RGO nanosheets have a large number of defects, oxygen-containing functional groups, and dangling bonds, which provide active sites for dipole polarization loss.

**For 1D structured CNF:** In our case, CNFs with controllable length and density would construct the tailorable conductive networks. As a result, the increasing conductive networks would effectively improve the conductive loss of the material.

**For 0D structured particle:** The heterogeneous interface between Fe_3_O_4_ nanoparticle and carbon shell are benefit to the interfacial polarization, resulting in the improvement of the dielectric loss. In addition, it should be noted that the introduction of Ni@CNF and Fe_3_O_4_@C would not only regulate the dielectric loss, but also offer a certain magnetic loss, leading to the improvement of the impedance matching.

Furthermore, the synergistic effects of these components are not only conducive to improving the microwave attenuation of the material, but also significantly adjust the impedance matching. **Frist,** the existence of helical CNCs would create the gaps and pores between the graphene layers, which lengthen the transmission distances of electromagnetic wave owing to the multiple reflect and scatter effects. Meanwhile, porous structure of aerogel ensures the growth space for CNFs, and facilitates the storage of air which would improve the impedance matching. **Second,** the absorbers with higher conductivity would lead to poor microwave absorption performance due to the skin effect. In this paper, the CNCs and CNFs are used to tailor the conductivity of the material. On the one hand, the helical CNCs with amorphous structures insert into graphene layers, resulting in the formation of point-to-surface contacts (CNC-graphene) instead of surface-to-surface contacts (graphene -graphene). Therefore, the evolution of contact form leads to the conductivity decrease. On the other hand, the conductivity of hierarchical aerogels is effectively controlled by tailoring the growth density and length of CNFs. As a result, the optimized aerogel has tailoring microwave absorption capacity and good impedance matching performance in almost the entire frequency range.

## Conclusion

In summary, the “3D helix–2D sheet–1D fiber–0D dot” hierarchical aerogels have been successfully synthesized by sequential processes of hydrothermal self-assembly and in-situ CVD growth method. In particular, the 2D graphene layers are uniformly intercalated by 3D helical CNCs, which endow the aerogel with abundant porous structure and better dielectric properties. Meanwhile, the CNCs and CNFs are successfully used to tailor the conductivity of the material. Besides, by adjusting the content of the magnetic particle and CVD reaction time, tunable electromagnetic parameters and excellent impedance matching, which play a crucial effect in the microwave absorption performance. The hierarchical GCA–M_0.2_–10 aerogel shows outstanding RL_min_ (− 55.1 dB) and width expansion EAB values (5.6 GHz) when the mass load in the wax matrix is only 15 wt%. Moreover, the RL_min_ value of GCA–M_0.3_–20 aerogel reaches − 71.5 dB at 9.5 GHz, and the corresponding EAB covers the whole X-band. The remarkable microwave absorption performance of the as-prepared aerogel is closely related to the multiple loss mechanisms of multi-dimensional gradient structures and the synergistic effect of each component. Our findings provide valuable guidance and inspiration for the design of multilevel hierarchical structure materials for microwave absorbing application and other related fields.

## Supplementary Information

Below is the link to the electronic supplementary material.

Supplementary file 1 (PDF 2117 KB)
