# Supplementary material for: Structural Engineering of Hierarchical Aerogels Comprised of Multi-dimensional Gradient Carbon Nanoarchitectures for Highly Efficient Microwave Absorption
Source: Nanomicro Lett. 2021 Jun 15;13:144. doi: 10.1007/s40820-021-00667-7 (PMC8206232; doi:10.1007/s40820-021-00667-7)
Supplement: Supplementary file 1 — (PDF 2117 KB) [file 40820_2021_667_MOESM1_ESM.pdf]

Supporting Infromation for

Structural Engineering of Hierarchical Aerogels Comprised of Multidimensional Gradient Carbon Nanoarchitectures for Highly Efficient Microwave Absorption

Yongpeng Zhao[1, 2], Xueqing Zuo[1], Yuan Guo[1], Hui Huang[1], Hao Zhang[1], Ting Wang[1], Ningxuan Wen[1], Huan Chen[1, 2], Tianze Cong[1], Javid Muhammad[1], Xuan Yang[3], Xinnan Wang[4], Zeng fan[1, *], Lujun Pan[1, *]

[1]School of Physics, Dalian University of Technology, Dalian, Liaoning 116024, P. R. China

[2]School of Microelectronics, Dalian University of Technology, Dalian, Liaoning 116024, P. R. China

[3]School of Materials Science and Engineering, Dalian University of Technology, Dalian, Liaoning 116024, P. R. China

[4]School of Chemical Engineering, Dalian University of Technology, Dalian, Liaoning 116024, P. R. China

*Corresponding authors. E-mail: lpan@dlut.edu.cn (L. Pan); fanzeng@dlut.edu.cn (Z. Fan)

Supplementary Tables and Figures

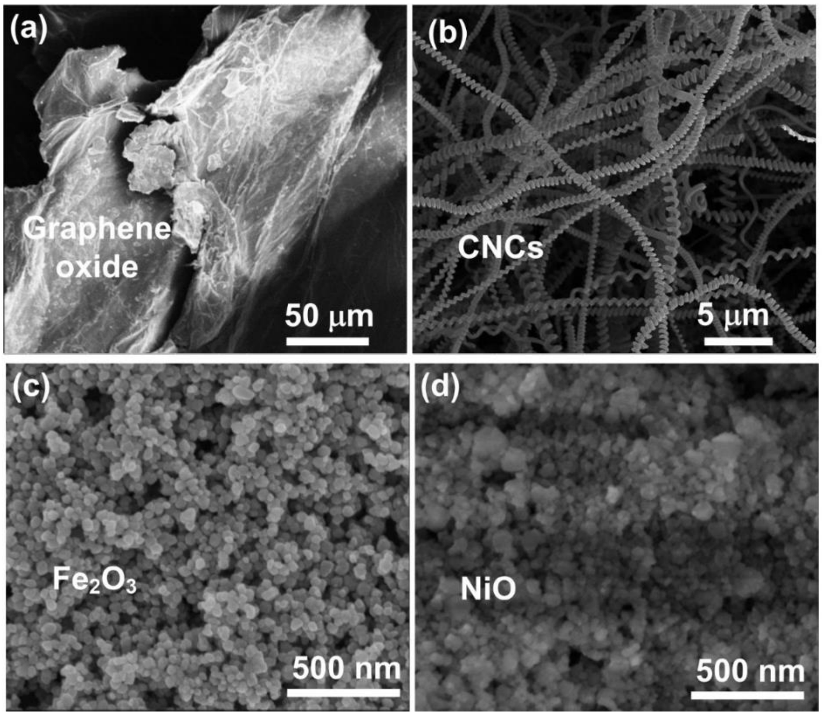

Fig. S1 SEM images of (**a**) graphene oxide, (**b**) CNCs, (**c**) Fe_2O_3, and (**d**) NiO

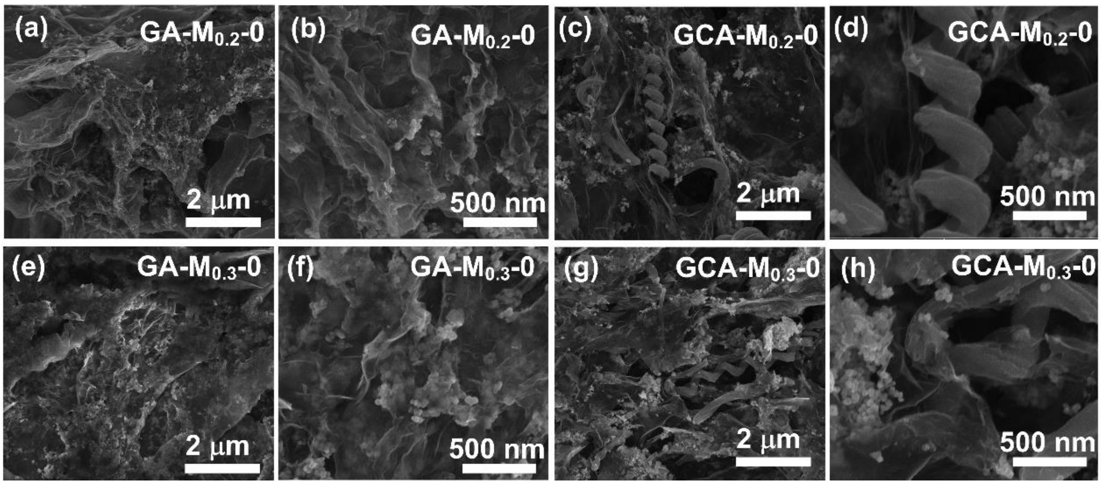

Fig. S2 SEM images of (**a, b**) GA-$M_{0.2}$-0, (**c, d**) GCA-$M_{0.2}$-0, (**e**, **f**) GA-$M_{0.3}$-0, and (**g**, **h**) GCA-$M_{0.3}$-0, respectively

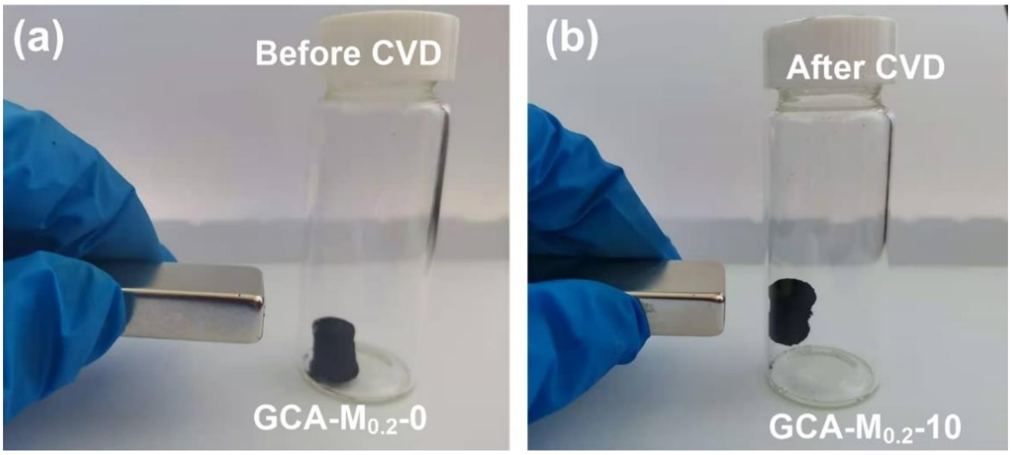

Fig. S3 Optical image of a GCA-$M_{0.2}$-0 aerogel before (**a**) and after (**b**) the CVD process

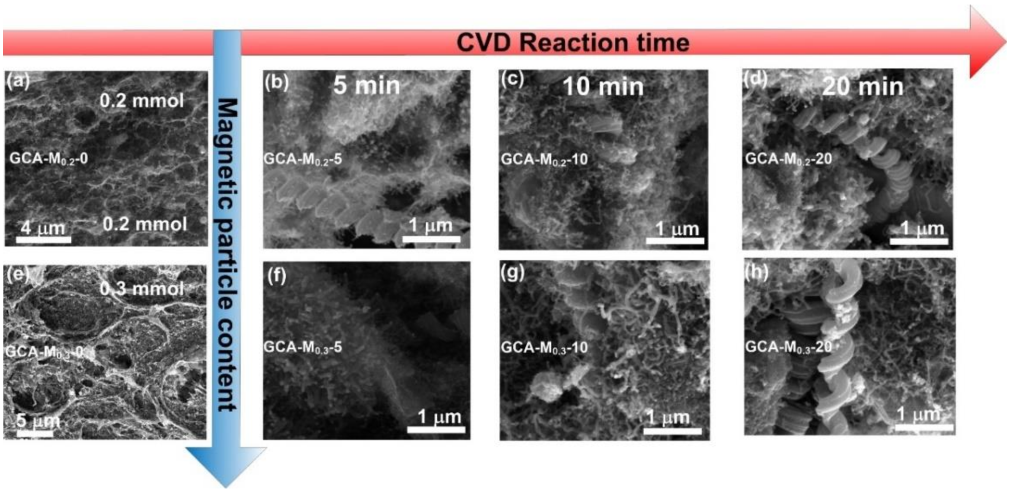

Fig. S4 SEM images of (**a**) GCA-$M_{0.2}$-0, (**b**) GCA-$M_{0.2}$-5, (**c**) GCA-$M_{0.2}$-10, (**d**) GCA-$M_{0.2}$-10, (**e**) GCA-$M_{0.3}$-0, (**f**) GCA-$M_{0.3}$-5, (**g**) GCA-$M_{0.3}$-10, and (**f**) GCA-$M_{0.3}$-20

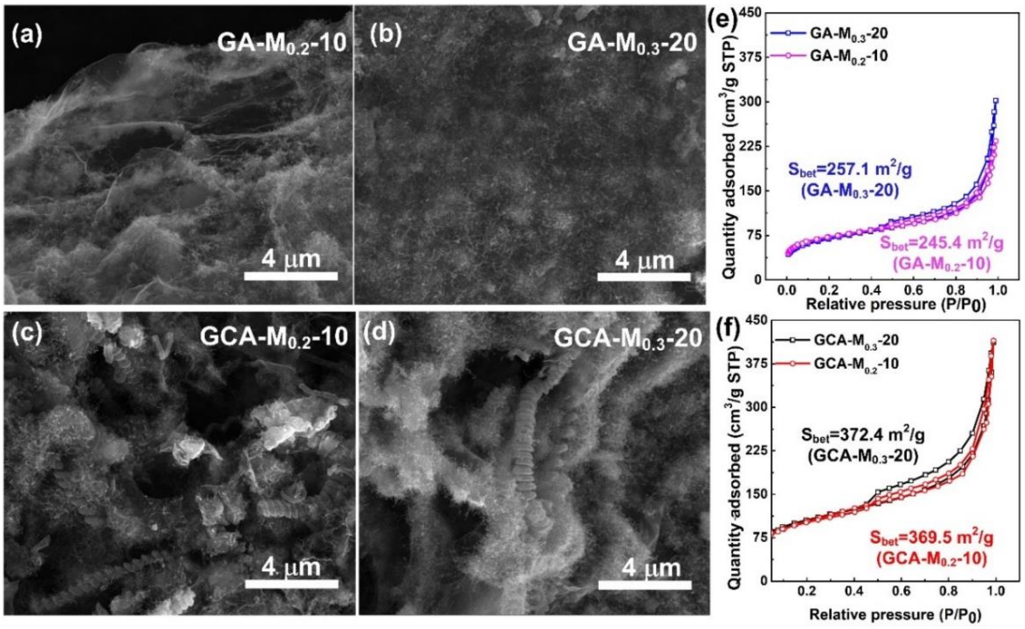

Fig. S5 SEM images of (**a**) GA-$M_{0.2}$-10, (**b**) GA-$M_{0.3}$-20, (**c**) GCA-$M_{0.2}$-10, and (**d**) GCA-$M_{0.3}$-20, respectively. The N_2 adsorption-desorption isotherms of (**e**) GA-$M_{0.2}$-10 and GA-$M_{0.3}$-20, (**f**) GCA-$M_{0.2}$-10 and GCA-$M_{0.3}$-20

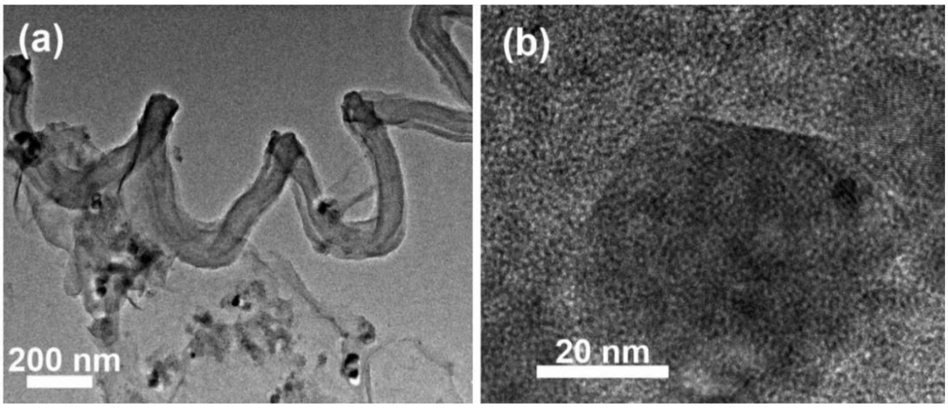

Fig. S6 (**a**) TEM and (**b**) TRTEM images of all the GCA-$M_{0.2}$-0 samples

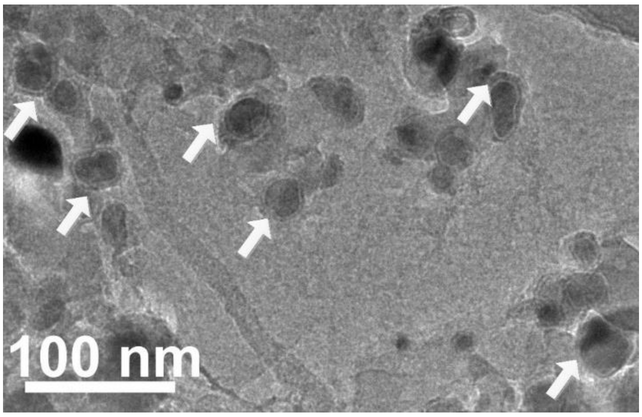

Fig. S7 Detailed TEM image of core-shell structured particles in GCA-$M_{0.3}$-20

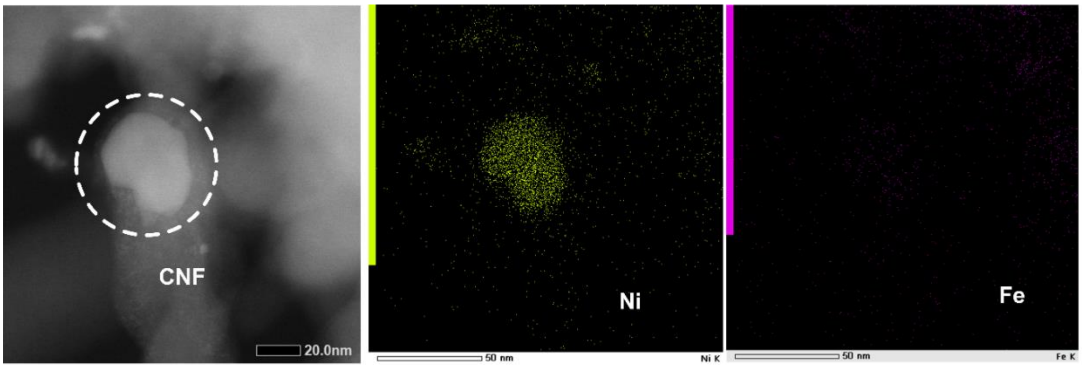

Fig. S8 Elemental maps of Ni and Fe in the tip of CNF

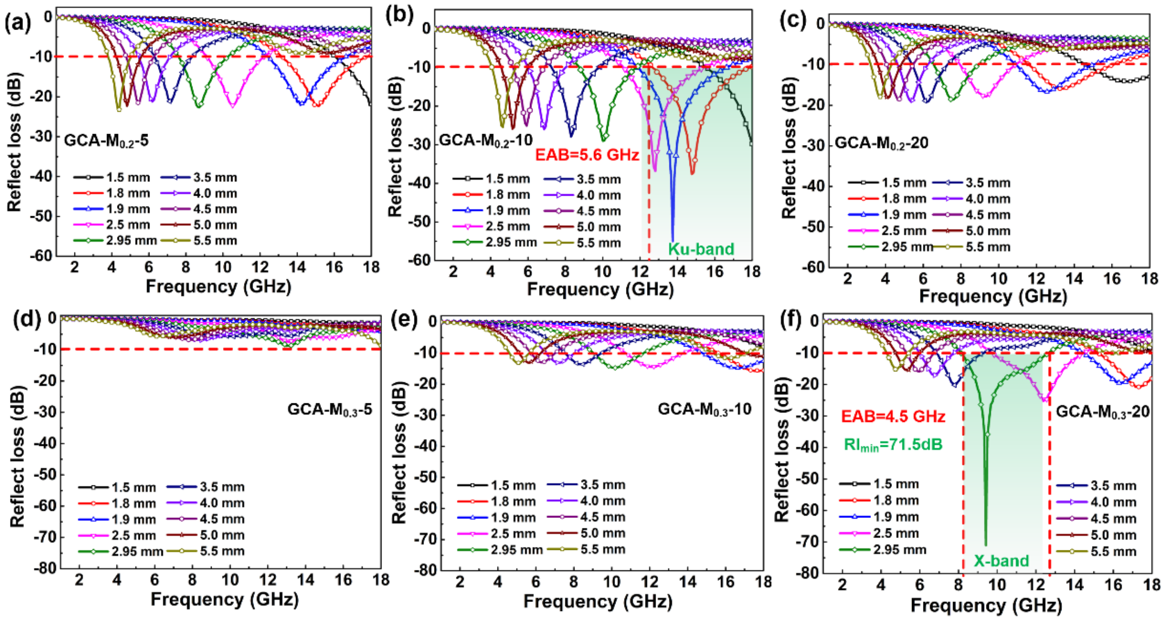

Fig. S9 Reflection loss values of (**a**) GCA-M$_{0.2}$-5, (**b**) GCA-M$_{0.2}$-10, (**c**) GCA-M$_{0.2}$-20, (**d**) GCA-M$_{0.3}$-5, (**e**) GCA-M$_{0.3}$-10, and (**f**) GCA-M$_{0.3}$-20

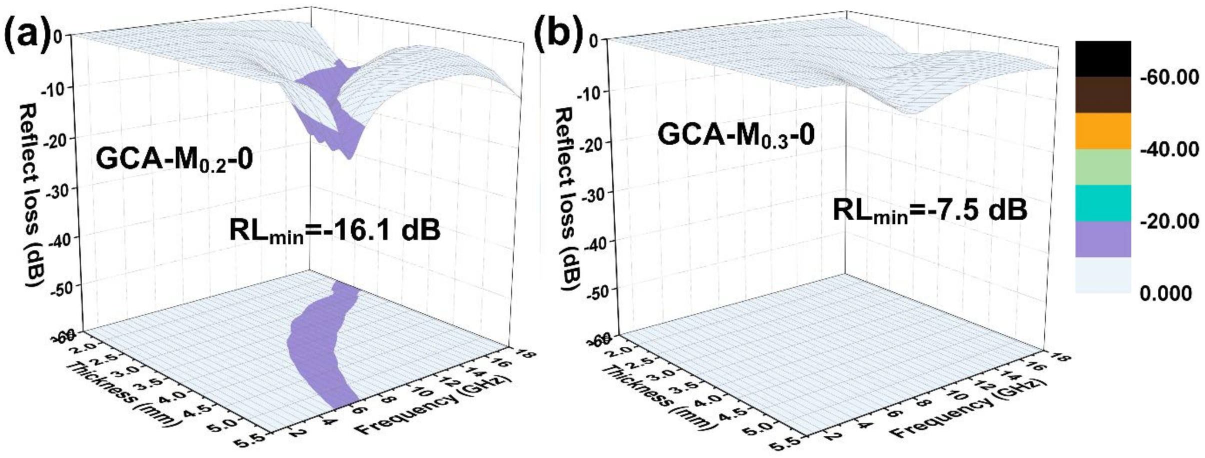

Fig. S10 3D representations of the RL of (**a**) GCA-M$_{0.2}$-0, (**b**) GCA-M$_{0.3}$-0

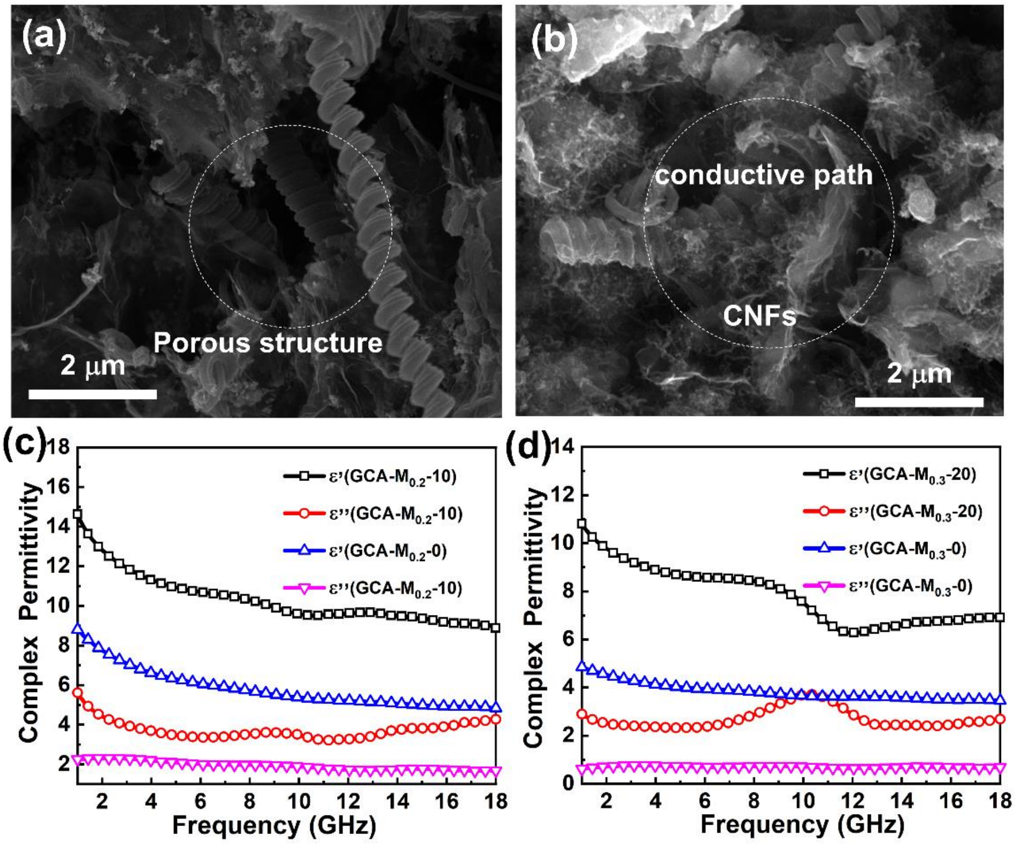

Fig. S11 SEM images of (**a**) GCA-M$_{0.2}$-0, (**b**) GCA-M$_{0.2}$-10; (**c**) Complex permittivity of GCA-M$_{0.2}$-0 and GCA-M$_{0.2}$-10; (**d**) Complex permittivity of GCA-M$_{0.3}$-0 and GCA-M$_{0.3}$-20

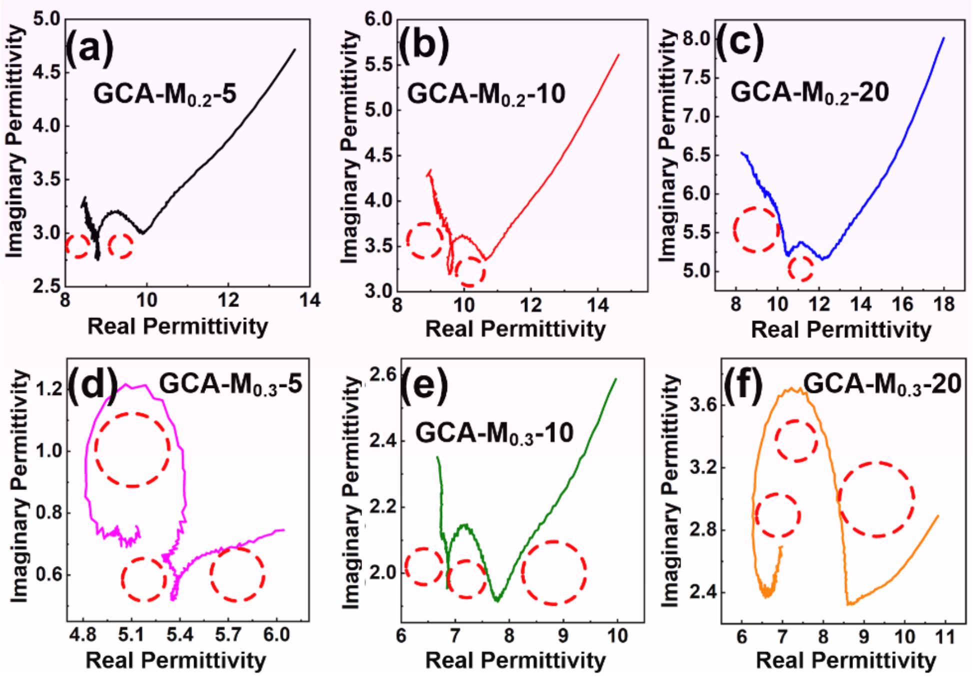

Fig. S12 Cole-Cole curves of (**a**) GCA-M$_{0.2}$-5, (**b**) GCA-M$_{0.2}$-10, (**c**) GCA-M$_{0.2}$-20, (**d**) GCA-M$_{0.3}$-5, (**e**) GCA-M$_{0.3}$-10, and (**f**) GCA-M$_{0.3}$-20

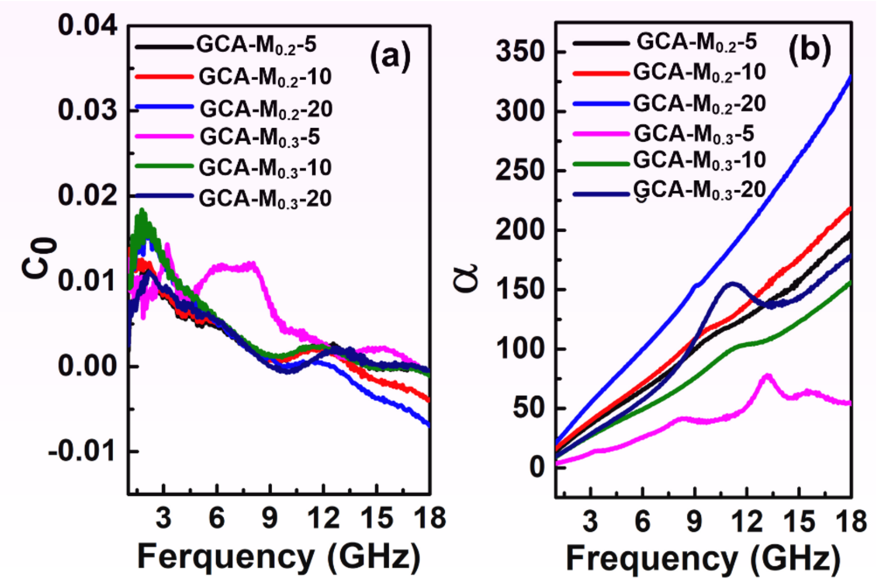

Fig. S13 Calculated C_0 (**a**) and a values (**b**) of as-obtained samples

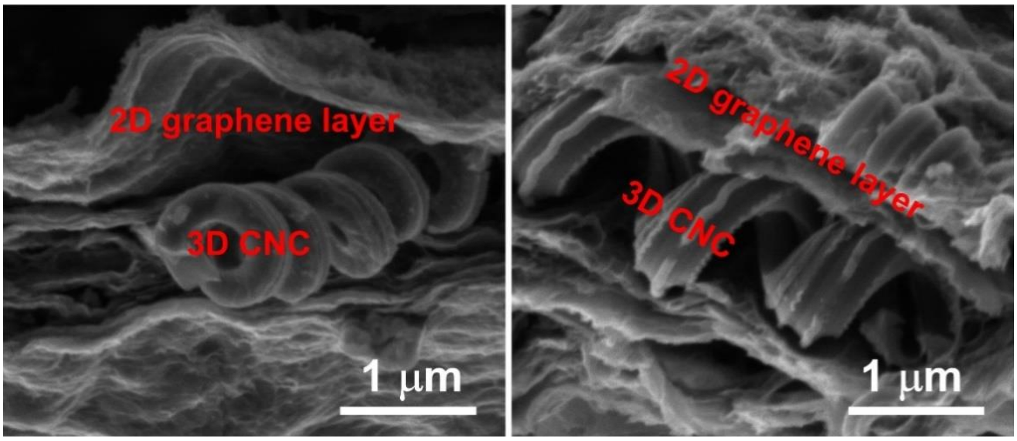

Fig. S14 Detailed SEM images of CNC and reduced graphene oxide existing in the RGO/CNC aerogel

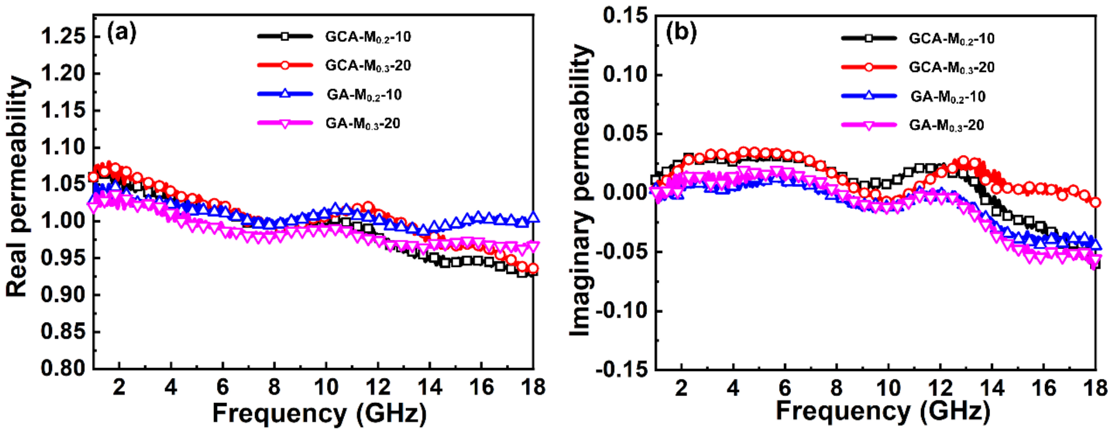

Fig. S15 (**a**) Real- and (**b**) imaginary- permeability for GCA-$M_{0.2}$-10-, GCA-$M_{0.3}$-20-, GA-$M_{0.2}$-10-, and GA-$M_{0.3}$-20-wax composites

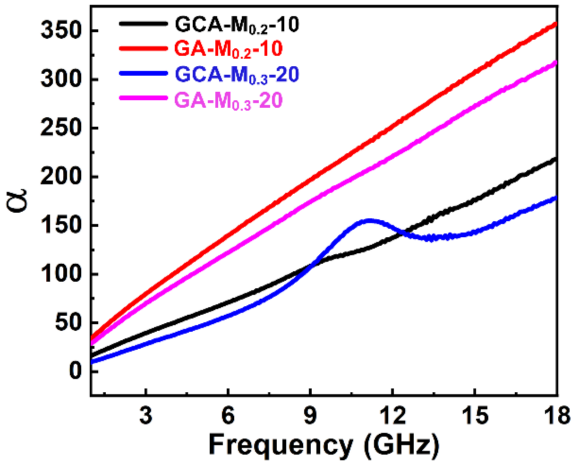

Fig. S16 Calculated attenuation constants of typical GA-M_X-Y and GCA-M_X-Y samples

Table S1 A comparison of specific surface for hierarchical RGO-based materials

Absorbers	Methods	Specific Surface Area(m^2/g)	Refs.
**Porous Fe_3O_4/rGO**	solvothermal	148	[S1]
**Fe_3O_4@SiO_2@MnO_2-RGO**	self-assembly	98.5	[S2]
**Nanoporous RGO-CNT**	self-assembly	231.4	[S3]
**Porous Graphene Microflowers**	spray-drying	230	[S4]
**CNF/rGO**	electrospinning	43.8	[S5]
**MoS_2@ CNT/RGO**	solvothermal	237	[S6]
**Co/Co_3O_4/CNTs/RGO**	solvothermal	194.6	[S7]
**nitrogen-doped rGO-CNT**	pyrolysis	125.29	[S8]
**β-FeOOH/rGO/CNT**	self-assembly	293.1	[S9]
**GCA-$M_{0.3}$-20**	self-assembly	372.4	This work
**GCA-$M_{0.2}$-10**	self-assembly	369.5	This work

Table 2 A comparison of microwave absorption performance of hierarchical carbon-based materials in X-band

Absorbers	Thickness (mm)	RL_{min} (dB)	EAB_{min} (GHz)	Filler loading	Refs.
**Graphene spheres-AC**	3.7	-32.4	4.2 (3.50 mm)	10 %	[S10]
**RGO-$NiZnFeO_4$**	2.91	-63	5.4 (2.00 mm)	40 %	[S11]
**RGO-SnO_2**	2.2	-53.7	3.7 (2.20 mm)	70 %	[S12]
**RGO-CNT-Fe_3O_4**	4	-49	4.1 (2.50 mm)	15 %	[S13]
**N-doped Graphene**	2.9	-43.2	5.0 (2.90 mm)	40 %	[S14]
**$CoFe_2O_4$-RGO**	2.1	-60.1	4.8 (2.20 mm)	20 %	[S15]
**PPY-SiC-RGO**	3	-22.5	4.4 (3.0 mm)	20 %	[S16]
**Co-C-MWCNTs**	2.5	-50	3.6 (2.5 mm)	25 %	[S17]
**N-doped Carbon aerogel**	2.6	-61.7	5.3 (2.6 mm)	20 %	[S18]
**GCA-$M_{0.3}$-20**	2.95	-71.5	4.5 (2.95 mm)	15 %	This work

Table S3 A comparison of microwave absorption performance of hierarchical carbon-based materials in Ku-band

Absorbers	Thickness (mm)	RL_{min} (dB)	EAB (GHz)	Filler loading	Refs.

**$CoFe_2O_4$-RGO**	2.1	-60.4	6.4 (2.2 mm)	20 %	[S15]
**MnO_2-SiC-RGO**	1.59	-54	7.4 (1.59 mm)	50 %	[S19]
**CNC-Fe_3O_4-C**	1.7	-47.5	5.0 (1.5 mm)	40 %	[S20]
**CNF-CNT-Co**	2.0	-52.3	5.1 (2.0 mm)	15 %	[S21]
**$LiFePO_4$-RGO**	2.4	-61.4	4.0 (2.4 mm)	30 %	[S22]
**RGO-CNT-FeNi**	2	-39.3	4.7 (1.8 mm)	10 %	[S23]
**GRO-PEG**	2.35	-43.2	5.3 (2.35 mm)	50 %	[S24]
**Biomass Carbon-Ni**	1.7	-52	5.0 (1.65 mm)	15 %	[S25]
**Co-CNTs-Carbon Sponge**	2.2	-51.2	4.1(2.2 mm)	10 %	[S26]
**FeCo-C**	2.8	-61.8	9.2 (2.8 mm)	10 %	[S27]
**CoNi/rGO**	0.8	-53	3.0 (0.8 mm)	7%	[S28]
**GCA-M$_{0.2}$-10**	1.9	-55.1	5.6 (1.8 mm)	15 %	This work

Supplementary References

[S1] Z. Liu et al., Rational design of hierarchical porous Fe_3O_4/RGO composites with lightweight and high-efficiency microwave absorption. Compos. Commun. **22**, 100492 (2020). https://doi.org/10.1016/j.coco.2020.100492

[S2] M. Ma et al., 1D flower-like Fe_3O_4@ SiO_2@ MnO_2 nanochains inducing RGO self-assembly into aerogels for high-efficient microwave absorption. Mater. Des. **188**, 108462 (2020). https://doi.org/10.1016/j.matdes.2019.108462

[S3] J.H. Kim et al., 3D-interconnected nanoporous RGO-CNT structure for supercapacitors application. Electrochim. Acta **125**, 536-542 (2014). https://doi.org/10.1016/j.electacta.2014.01.142

[S4] C. Chen et al., Porous graphene microflowers for high-performance microwave absorption. Nano-Micro Lett. **10**(2), 26 (2018). https://doi.org/10.1007/s40820-017-0156-2

[S5] S.H. Han et al., High areal capacity of Li-S batteries enabled by freestanding CNF/rGO electrode with high loading of lithium polysulfide. Electrochim. Acta **241**, 406-413 (2017). https://doi.org/10.1016/j.electacta.2017.05.005

[S6] S.H. Wang et al., Three dimensional MoS_2@ CNT/RGO network composites for high performance flexible supercapacitors. Chem. Eur. J. **23**(14), 3438-3446 (2017). https://doi.org/10.1002/chem.201605465

[S7] Y. Wang et al., MOF-derived nanoporous carbon/Co/Co_3O_4/CNTs/RGO composite with hierarchical structure as a high-efficiency electromagnetic wave absorber. J. Alloys Compd. **846**, 156215 (2020). https://doi.org/10.1016/j.jallcom.2020.156215

[S8] C.H. Guo et al., Space-confined iron nanoparticles in a 3D nitrogen-doped rGO-CNT framework as efficient bifunctional electrocatalysts for rechargeable Zinc–Air batteries. Micropor. Mesopor. Mat. **298**, 110100 (2020). https://doi.org/10.1016/j.micromeso.2020.110100

[S9] S.W. Bokhari et al., Self-assembly of β-FeOOH/rGO/CNT for a high-

performance supercapacitor. Mater. Lett. **220**, 140-143 (2018). https://doi.org/10.1016/j.matlet.2018.02.131

[S10] H.L. Xu et al., Constructing hollow graphene nano-spheres confined in porous amorphous carbon particles for achieving full X band microwave absorption. Carbon **142**, 346-353 (2019). https://doi.org/10.1016/j.carbon.2018.10.056

[S11] R.W. Shu et al., Facile synthesis of nitrogen-doped reduced graphene oxide/nickel-zinc ferrite composites as high-performance microwave absorbers in the X-band. Chem. Eng. J. **384**, 123266 (2020). https://doi.org/10.1016/j.cej.2019.123266

[S12] J.B. Zhang et al., Facile fabrication and enhanced microwave absorption properties of reduced graphene oxide/tin dioxide binary nanocomposites in the X-band. Synth. Met. **257**, 116157 (2019). https://doi.org/10.1016/j.synthmet.2019.116157

[S13] Y. Qin et al., Preparation of graphene aerogel with high mechanical stability and microwave absorption ability via combining surface support of metallic-CNTs and interfacial cross-linking by magnetic nanoparticles. ACS Appl. Mater. Interfaces **11**(10), 10409-10417 (2019). https://doi.org/10.1021/acsami.8b22382

[S14] Y.L. Zhou et al., Graphene nanoflakes with optimized nitrogen doping fabricated by arc discharge as highly efficient absorbers toward microwave absorption. Carbon **148**, 204-213 (2019). https://doi.org/10.1016/j.carbon.2019.03.034

[S15] X.Y. Wang, et al., $CoFe_2O_4$/N-doped reduced graphene oxide aerogels for high-performance microwave absorption. Chem. Eng. J. **388**, 124317 (2020). https://doi.org/10.1016/j.cej.2020.124317

[S16] Y.H. Cheng et al., Achieving tunability of effective electromagnetic wave absorption between the whole X-band and Ku-band via adjusting PPy loading in SiC nanowires/graphene hybrid foam. Carbon **132**, 430-443 (2018). https://doi.org/10.1016/j.carbon.2018.02.084

[S17] R.W. Shu et al., Nitrogen-doped Co-C/MWCNTs nanocomposites derived from bimetallic metal–organic frameworks for electromagnetic wave absorption in the X-band. Chem. Eng. J. **362**, 513-524 (2019). https://doi.org/10.1016/j.cej.2019.01.090

[S18] P.B. Liu et al., Vacancies-engineered and heteroatoms-regulated N-doped porous carbon aerogel for ultrahigh microwave absorption. Carbon **169**, 276-287 (2020). https://doi.org/10.1016/j.carbon.2020.07.063

[S19] S. Dong et al., SiC whiskers-reduced graphene oxide composites decorated with MnO nanoparticles for tunable microwave absorption. Chem. Eng. J. **392**, 123817 (2020). https://doi.org/10.1016/j.cej.2019.123817

[S20] Y.P. Zhao et al., In situ construction of hierarchical core–shell Fe_3O_4@C nanoparticles–helical carbon nanocoil hybrid composites for highly efficient electromagnetic wave absorption. Carbon **171**, 395-408 (2021). https://doi.org/10.1016/j.carbon.2020.09.036

[S21] Z.C. Wu et al., Enhanced microwave absorption performance from magnetic coupling of magnetic nanoparticles suspended within hierarchically tubular composite. Adv. Funct. Mater. **29**(28), 1901448 (2019). https://doi.org/10.1002/adfm.201901448

[S22] J.J. Dong et al., Three-dimensional architecture reduced graphene oxide–$LiFePO_4$ composite: preparation and excellent microwave absorption performance. Inorg. Chem. **58**(3), 2031-2041 (2019). https://doi.org/10.1021/acs.inorgchem.8b03043

[S23] J. Xu et al., N-doped reduced graphene oxide aerogels containing pod-like N-doped carbon nanotubes and FeNi nanoparticles for electromagnetic wave absorption. Carbon **159**, 357-365 (2020). https://doi.org/10.1016/j.carbon.2019.12.020

[S24] H.M. Ji et al., Remarkable microwave absorption performance of ultralight graphene-polyethylene glycol composite aerogels with a very low loading ratio of graphene. Compos. Part A: Appl. Sci. Manufac. **123**, 158-169 (2019). https://doi.org/10.1016/j.compositesa.2019.05.012

[S25] H.Q. Zhao et al., A novel hierarchically porous magnetic carbon derived from biomass for strong lightweight microwave absorption. Carbon **142**, 245-253 (2019). https://doi.org/10.1016/j.carbon.2018.10.027

[S26] N. Yang et al., Ultralight three-dimensional hierarchical cobalt nanocrystals/N-doped CNTs/carbon sponge composites with a hollow skeleton toward superior microwave absorption. ACS Appl. Mater. Interfaces **11**(39), 35987-35998 (2019). https://doi.org/10.1021/acsami.9b11101

[S27] L. Wang et al., Hierarchical nest-like structure of Co/Fe MOF derived CoFe@ C composite as wide-bandwidth microwave absorber. Compos. Part A: Appl. Sci. Manufac. **135**, 105958 (2020). https://doi.org/10.1016/j.compositesa.2020.105958

[S28] H.-B. Zhao, J.-B. Cheng, J.-Y. Zhu, Y.-Z. Wang, Ultralight CoNi/rGO aerogels toward excellent microwave absorption at ultrathin thickness. J. Mater. Chem. C **7**(2), 441-448 (2019). https://doi.org/10.1039/C8TC05239E
